# Digital Interventions Targeting Healthy and Sustainable Eating Behavior: Systematic Review and Meta-Analysis

**DOI:** 10.2196/80821

**Published:** 2026-01-08

**Authors:** Käbi Vanwinkelen, Bram Spruyt, Tim Smits

**Affiliations:** 1 Media, Information and Persuasion Lab, Department of Communication Science, KU Leuven, Parkstraat 45, Leuven, 3000, Belgium, 32 016326735

**Keywords:** diet, healthy eating, digital media, social media, best practices, internet-based intervention, nutrition intervention, digital interventions, systematic review, meta-analysis

## Abstract

**Background:**

Current food consumption patterns contribute to the rising prevalence of obesity and noncommunicable diseases and exacerbate environmental degradation. Digital media offer promising opportunities to promote healthier and more sustainable eating; yet, evidence regarding their effectiveness remains fragmented.

**Objective:**

The aim of this systematic review and meta-analysis is (1) to evaluate the effectiveness of digital interventions in improving healthy and sustainable food consumption and (2) to identify which participant and intervention characteristics are associated with greater effectiveness.

**Methods:**

A systematic search was conducted in January 2024 and repeated in September 2025 across Web of Science, Embase, and Scopus, supplemented with forward and backward reference searching. Eligible studies were those with a quasi-experimental or longitudinal design evaluating digital interventions targeting nonclinical populations, with the aim of increasing plant-based food consumption or reducing animal-based food intake. Risk of bias was assessed using the Cochrane risk-of-bias tool. Included interventions were coded for behavior change techniques using the Behavior Change Taxonomy version 1. A random-effects meta-analysis with robust variance estimation was performed, and moderator analyses were conducted with participant and intervention characteristics.

**Results:**

Eligibility screening led to the inclusion of 52 papers published between 2004 and 2025, with 24,652 participants in total. The meta-analysis revealed a small but statistically significant positive effect of digital interventions on food consumption outcomes (*d*=0.33, 95% CI 0.25‐0.42; *P<*.001). However, substantial heterogeneity (*I*^2^*=*86%, 95% prediction interval −0.21 to 0.87) indicates considerable variation in effectiveness across intervention characteristics. A moderator analysis showed no significant difference in effectiveness (*P*=.53) between interventions aimed at reducing meat consumption (*d*=0.38, 95% CI 0.20‐0.57; *P*<.001) and those promoting plant-based eating (*d*=0.33, 95% CI 0.23‐0.42; *P*<.001). Although digital interventions had the strongest effects among young adults (*d*=0.46, 95% CI 0.30‐0.61; *P*<.001), age-related differences were not statistically significant. Intervention effectiveness differed significantly by platform (*P*=.03), with social media interventions (*d*=0.65, 95% CI 0.41‐0.90; *P*<.001) yielding stronger effects than other modalities. Incorporating prompts or cues significantly enhanced effectiveness (*d*=0.58 vs *d*=0.30; *P*=.04). Although not statistically significant, interventions including social support or behavioral comparison (both *d*=0.39; *P*<.001) yielded larger effects. Few studies included adolescents or individuals from lower socioeconomic backgrounds.

**Conclusions:**

This review underscores the innovative potential of digital interventions in improving eating behavior, highlighting how effectiveness varies by intervention design. Social media emerge as particularly promising, likely due to their unique social and interactive features. By pinpointing the contexts and types of digital interventions that most effectively promote plant-based eating, this study provides timely guidance for researchers and practitioners in increasingly digitalized food environments. Nonetheless, more high-quality studies are needed to confirm these insights and address the critical gap among adolescents and low socioeconomic groups.

## Introduction

Food plays a pivotal role in both human and planetary health. Current food consumption patterns are driving the steep increase in obesity and noncommunicable diseases such as diabetes or cancers [1]. Simultaneously, contemporary dietary habits contribute significantly to greenhouse gas emissions, deforestation, and water scarcity, thereby exacerbating environmental degradation [2]. As high intake of animal-based foods plays a substantial role herein, the EAT-Lancet Commission emphasizes the urgency of a worldwide shift to healthy and sustainable diets mainly characterized by a variety of plant-based foods (eg, fruits and vegetables, whole grains, and legumes) and low quantities of animal-based foods [2,3]. Adherence to the EAT-Lancet diet is associated with a lower risk of diabetes, cardiovascular disease, and cancer-related mortality while reducing greenhouse gas emissions by 29% [4-6]. The most profound health and environmental benefits can be achieved by reducing meat consumption and increasing the intake of fruit, vegetables, and legumes [7-9]. Nevertheless, global meat consumption has risen substantially over the past 5 decades and is projected to continue increasing, while intake of fruits, vegetables, and legumes remains inadequate [10-13]. Hence, it is crucial to explore effective strategies for promoting plant-based dietary patterns.

The widespread use of digital media and their capacity to influence consumption patterns through the promotion of unhealthy foods have sparked growing interest among researchers in leveraging them for health interventions [14,15]. Digital interventions can be delivered through a variety of platforms, including mobile apps, SMS text messaging, websites, and perhaps, most notably, social media. While digital intervention studies often still rely on custom-built or more traditional platforms, health researchers are increasingly exploring the potential of social media for dietary interventions and health promotion campaigns [16-19]. These developments highlight the importance of evaluating and comparing the effectiveness of different digital platforms for interventions [20,21].

To achieve dietary behavior change, interventions incorporate behavior change techniques (BCTs) [22]. BCTs are the smallest identifiable components of an intervention that can independently influence behavior [23,24]. Strategically implementing BCTs has been consistently emphasized as essential for developing effective interventions [22,25]. However, it remains unclear which techniques are most effective in improving healthy and sustainable eating and which combinations of BCTs and digital intervention platforms enhance the intervention’s effectiveness. Hence, systematically reviewing and identifying the BCTs most strongly associated with the intervention’s effects could enhance the design of future programs targeting food consumption [23].

Although digital interventions targeting eating behavior are gaining popularity, prior reviews report mixed conclusions regarding their effectiveness [18,19,26-29]. Notably, many of these reviews were narrow in scope: some focused on specific food categories, such as sugar-sweetened beverages or vegetables [19,30,31], targeted specific age groups [19,32,33], or selectively included specific digital platforms [18,19,33,34]. Moreover, previous systematic reviews that summarize the literature on digital interventions often overlook social media and focus on research-created platforms or (now) outdated digital tools [16,17,19,35]. Systematic reviews that do consider social media use limited keywords in their search strategy or focus exclusively on social media platforms, missing the opportunity to compare traditional digital interventions with social media interventions [18,28,36,37]. In addition, few reviews assess which BCTs are most successful in targeting dietary change through digital interventions, nor do they explore whether effectiveness differs across age groups or socioeconomic groups [38]. This is critical, as individuals with lower socioeconomic status (SES) are disproportionately affected by poor diets, and the interventions that succeed in high SES populations may not be equally effective for low-income groups [19,39].

While narrow reviews are valuable for exploring targeted questions in depth, there is a need for a comprehensive synthesis that compares intervention strategies across different digital media to gain a more profound understanding of the key components that contribute to a successful digital intervention. Therefore, the aim of this systematic review and meta-analysis is (1) to evaluate the overall effectiveness of digital interventions in terms of increased healthy or sustainable eating behavior and (2) to identify when interventions are (more) effective by considering the behavioral goal orientation (prevention vs promotion approach), intervention characteristics (ie, the digital platform and BCTs), and participant characteristics (ie, age and SES).

## Methods

The reporting of this systematic review is in accordance with the PRISMA (Preferred Reporting Items for Systematic Reviews and Meta-Analyses) and PRISMA-S (Preferred Reporting Items for Systematic Reviews and Meta-Analyses Literature Search Extension) guidelines (Checklist 1) [40,41], and its protocol was prospectively registered in PROSPERO (CRD42023487955). This study was a secondary analysis of published literature and did not require ethics approval.

### Data Sources and Search Strategy

Systematic searches were conducted on January 19, 2024, in Web of Science core collection, Embase, and Scopus, with an updated search run on September 23, 2025, to retrieve newly published papers. An experienced academic librarian was consulted to optimize the search strategy and to ensure that database-specific operators were used correctly. The final search string was limited to peer-reviewed papers published in English and contained keywords related to digital media, interventions, and healthy and sustainable eating behavior (Multimedia Appendix 1). No date restrictions or other published search filters were applied. The reference lists of all included papers were manually searched, and a citation search using Web of Science was conducted. Authors of all included studies were contacted for additional relevant papers. No other online resources or study registries were searched.

### Eligibility Criteria

Studies were included if they met the following PICO-based criteria. Reviews, dissertations, books, and conference papers were excluded.

#### Population

Studies had to recruit a nonclinical population of participants. Studies exclusively including individuals with specific health conditions or specific nutritional needs (eg, diabetes, pregnancy, and eating disorders) were excluded. Similarly, populations primarily composed of individuals with overweight or obesity were not considered because of differences in appetite [42].

#### Intervention

Eligible studies had to test a digital intervention designed to promote healthy and sustainable eating behavior, specifically by increasing plant-based food consumption or reducing intake of animal-based products. An intervention was defined as “a coordinated set of activities designed to change specified behavior patterns” [43], thus excluding interventions with a one-time exposure, as these rarely allow for sustained behavioral changes. Multicomponent interventions with an offline element were not included, as effects (or lack thereof) cannot be specifically attributed to the digital component.

#### Comparison

Studies were required to include a form of baseline comparison or control group that could either be inactive (ie, no intervention and waitlist) or active (ie, alternative intervention). Studies had to use a quasi-experimental (eg, randomized controlled trial [RCT]) or longitudinal design.

#### Outcomes

Eligible studies had to provide an outcome measure related to eating behavior, such as food consumption, intention, choice, or purchase. These outcomes could be related to intake of plant-based foods (eg, fruits, vegetables, and legumes) and animal-based foods (eg, red or processed meat) or adherence to a healthy and sustainable diet (eg, Mediterranean diet).

### Data Extraction

KV and BS independently extracted relevant data using the preregistered extraction book, covering study characteristics (eg, authors, year of publication, and country of origin), study design (eg, comparator and research question), population (eg, age, gender, and SES indicators), intervention characteristics (eg, digital medium, duration, BCTs, and approach), outcomes (eg, behavioral outcomes, metrics, and covariates), and results (eg, analysis technique and statistical results). The 3 reviewers collaborated closely to resolve any discrepancies during the data extraction process.

### Coding of BCTs

BCTs that were not explicitly reported in the included studies were coded by 2 reviewers (KV and BS) using the Behavior Change Technique Taxonomy version 1 (BCTTv1) [24]. One reviewer (KV) coded all studies, and a second reviewer (BS) randomly double-coded 5, achieving 90% agreement. Both reviewers were certified coders, as they completed the online taxonomy training (“BCTTv1 Online Training”). Discrepancies were resolved via discussion between the 2 reviewers. For studies with multiple intervention groups (IG), BCTs were coded separately for each active arm. When insufficient detail was provided to code the BCTs, related documents (eg, protocols) were consulted.

### Risk of Bias Assessment

Included studies were appraised for study quality using the Cochrane risk-of-bias tool [44]. For RCTs, the Revised RoB 2 tool was used; for cluster RCTs, an adapted version of RoB 2; and for nonrandomized studies (NRS), ROBINS-I [45-47]. The risk of bias assessment was conducted with consideration of best practices in food and communication research. While self-reported measures typically carry a moderate risk of bias, this was not considered problematic, as self-reporting is often the most appropriate or even the only feasible method for measuring eating behavior.

**Figure 1.**
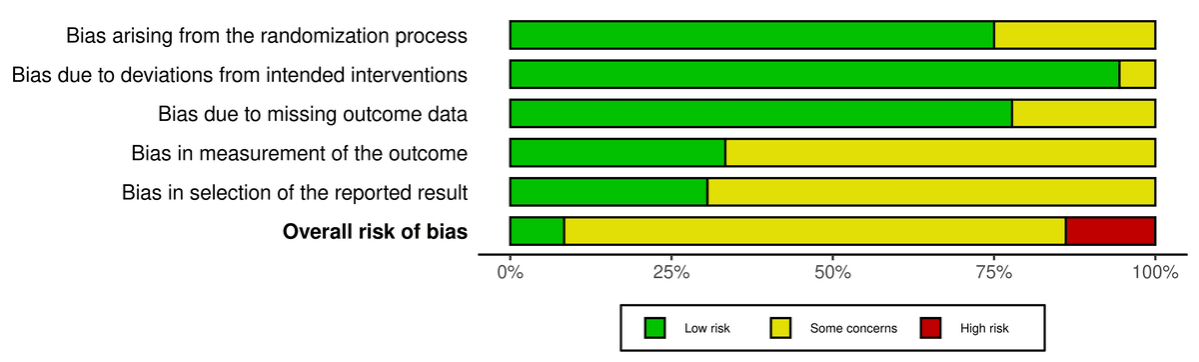
Visualization of the risk of bias of randomized controlled trials (n=36).

**Figure 2.**
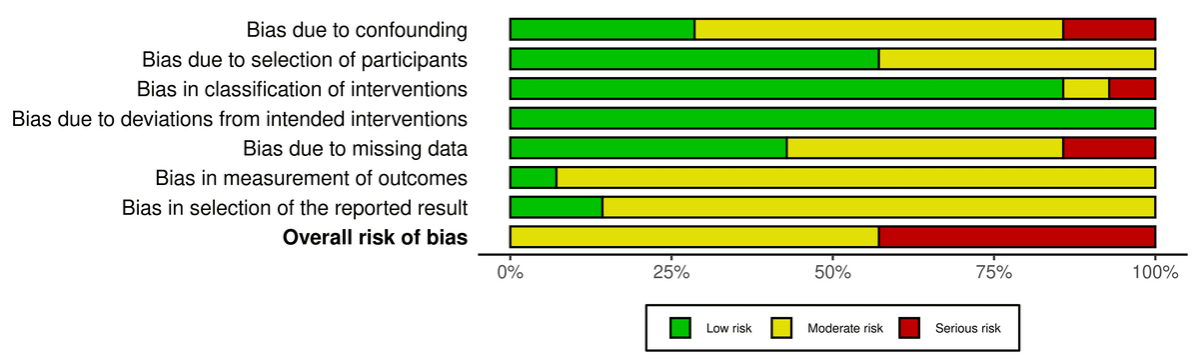
Visualization of the risk of bias of nonrandomized studies (n=14).

Risk of bias assessments were independently conducted by 2 reviewers (KV and BS), and discrepancies were resolved through consultation with a third reviewer (TS). One paper included 2 distinct experimental studies [48], resulting in a total of 53 separate studies evaluated for risk of bias. The majority of RCTs, including all 3 cluster RCTs, had a moderate risk of bias, primarily due to the use of self-report measures or lack of prespecified analysis plan (31/39, 79%; Figure 1) [49]. In total, 5 RCTs were rated as high risk and 3 as low risk. NRS were rated as moderate (8/14, 57%) or high (6/14, 43%) risk (Figure 2), commonly due to baseline confounding and issues similar to those in the RCTs (see Multimedia Appendix 2 [48,50-99] for a detailed overview of the risk of bias assessments).

### Data Synthesis

To narratively summarize the findings, IGs with differing content were treated as distinct, and identical interventions across studies were grouped [ie, 50-53]. A study was considered to have a consistent effect if it demonstrated a significant improvement in food consumption (ie, within-subjects effect) or if this change was significant compared to a control group (ie, between-subjects effect). For quantitative synthesis, standardized mean differences (Cohen *d*) were calculated. For pre- and postdesigns, Cohen *dav* was estimated, which is based on the mean difference and average SD of both sets of observations [100,101]. For studies that included both an intervention and control group, we calculated the effect size based on the mean pre- and postchange in the treatment group minus the mean pre- and postchange in the control group, divided by the pooled pretest SD [102]. This estimation has proven to be the most recommended effect size for repeated-measure designs in terms of bias and precision [103]. The variance of this effect size was calculated in R (R Foundation for Statistical Computing) with equation 25 of Morris [103], assuming a standard pre- to postintervention correlation of *r*=0.50. As an estimate of ρ (ie, the correlation of effect sizes) is required to develop efficient weights, several sensitivity analyses were conducted with correlation values of *r*=0.10 and *r*=0.90, which led to the standard size of *r*=0.5 [104]. Missing data were requested from authors and obtained for 2 papers.

A random-effects meta-analysis was performed, as we aimed to estimate the mean of a distribution of effects, and we anticipated heterogeneity in true effect sizes across studies due to variations in study characteristics [105]. Positive effect sizes reflect improvements in healthy or sustainable food intake (ie, increased plant-based consumption or reduced meat consumption), whereas negative values reflect declines. Effect sizes were dependent, as studies often contained multiple IGs compared to a common control, multiple types of food outcomes, or multiple follow-ups. Including effect sizes from the same study in a single model creates complications due to statistical dependence, which violates the assumption of independent sampling errors. To account for this dependency, robust variance estimation was performed in R with the packages *metafor*, *meta*, and *ClubSandwich* [106,107]. This method offers a robust solution to handle dependency, even when the nature of the dependence structure is unknown, by grouping effect sizes based on commonalities (ie, hierarchical clustering) and accounting for the correlation of sampling errors [107].

In total, 6 outliers and 1 intervention with inconsistent results were excluded, leaving 41 papers containing 57 interventions, with 82 effect sizes. Given that NRS represent an important part of the evidence base in digital intervention research and the lack of clear consensus on how to best integrate different types of study designs into meta-analyses [108,109], both RCTs and NRS were included in the meta-analyses. To assess the robustness of the findings, we conducted sensitivity analyses excluding all NRS. The modified method of Hartung-Knapp-Sidik-Jonkman was applied for greater accuracy [110]. Heterogeneity was evaluated using the *I*^2^ statistic and prediction intervals (PIs). Although *I*² is a commonly reported metric, researchers have noted that it may not be the most informative indicator of heterogeneity; PIs are often preferred, as they reflect the distribution of true effects [111,112]. Funnel plots and Egger test were applied to examine small-study effects; however, the interpretation of funnel plots should be approached with caution due to their known limitations [113]. To identify the conditions under which digital interventions differ in effectiveness, we conducted a series of exploratory moderator (ie, subgroup) analyses. More specifically, subgroup analyses were conducted to examine (1) the stability of effects (ie, postintervention vs follow-up), as well as variations in effectiveness based on (2) behavioral goal orientation, (3) participant age, (4) digital medium, and (5) the presence of each distinct BCT cluster. Each moderator was examined in a separate model to avoid overfitting. Moderator analyses were also conducted for individual BCTs, but results are reported only in Multimedia Appendix 3 due to the limited number of studies per BCT and their similarity to the findings from BCT cluster analyses. More information on the meta-analysis can be found in Multimedia Appendix 4 [50,51,78,96,100-107,114].

## Results

### Study Selection

**Figure 3.**
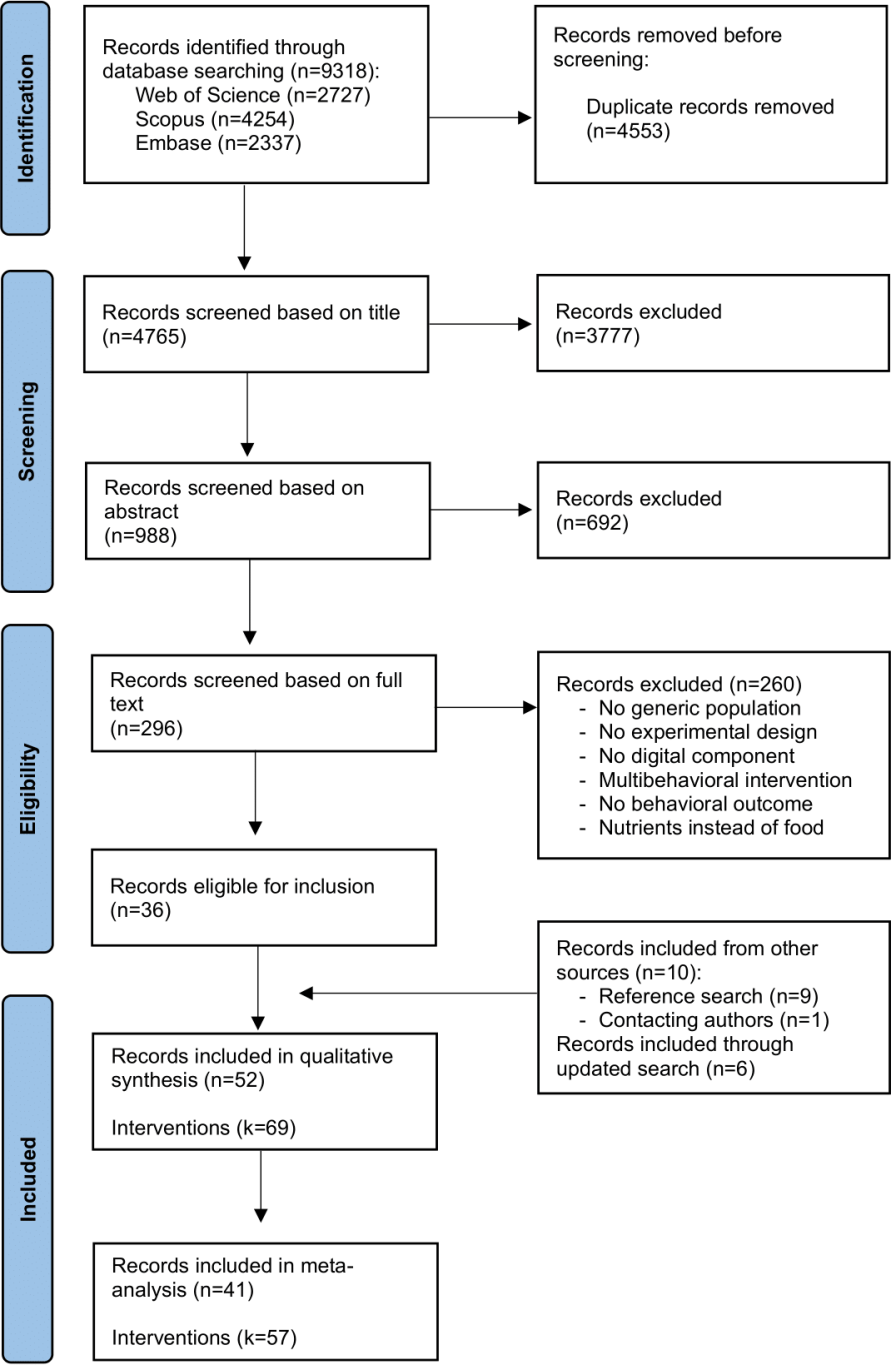
PRISMA (Preferred Reporting Items for Systematic Reviews and Meta-Analyses) flow diagram of study inclusion.

**Figure 4.**
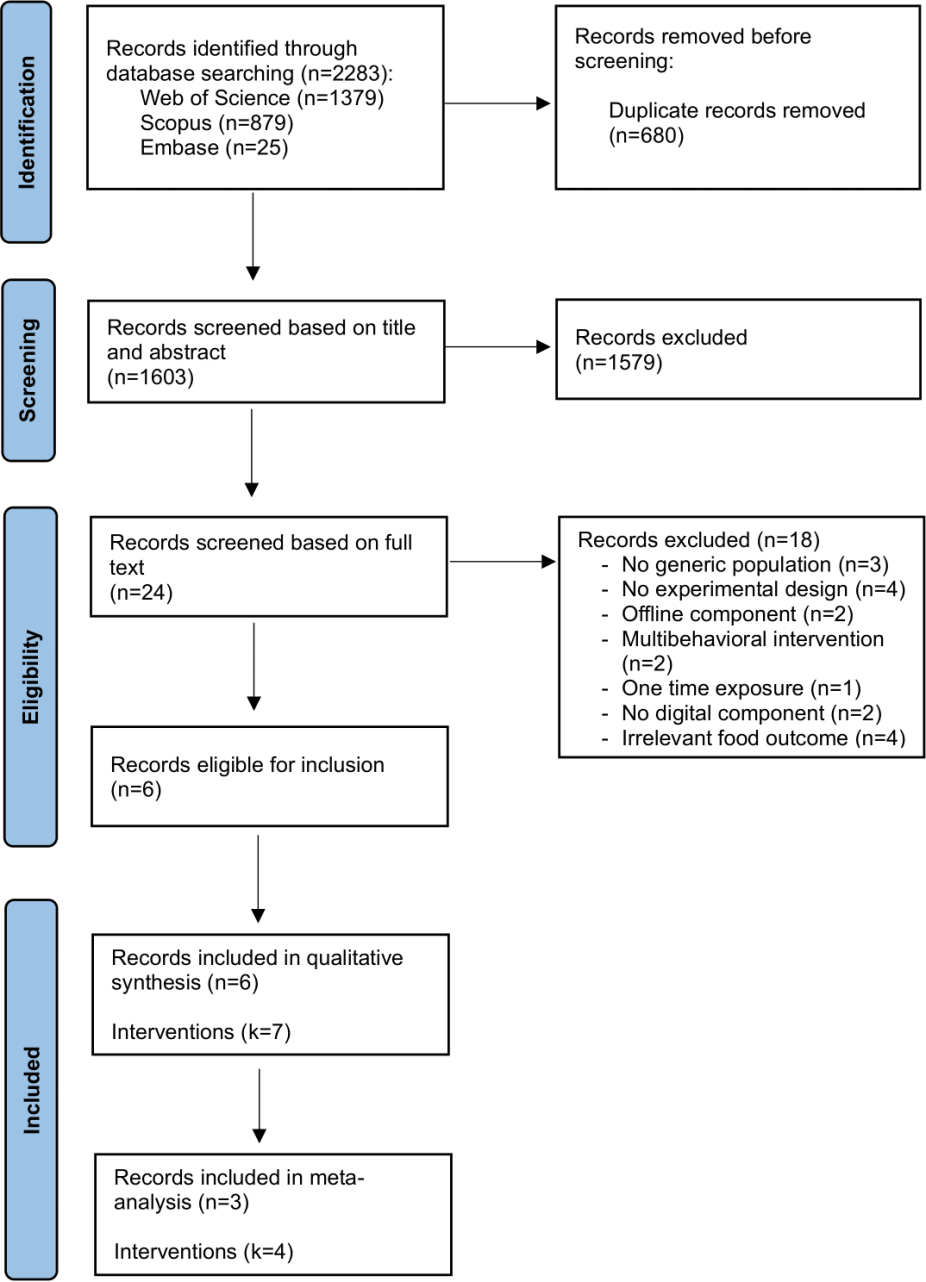
PRISMA (Preferred Reporting Items for Systematic Reviews and Meta-Analyses) flow diagram of study inclusion for updated search (September 23, 2025).

The database searches identified 9318 records in total. Following the removal of duplicates through the SR Accelerator software (Bond University) [115], 4765 papers were uploaded to Zotero for eligibility screening. Titles and abstracts were screened independently by the first author (KV) and 2 graduate students, followed by full-text screening. Papers that were commonly included by all screeners received a definitive inclusion. Discrepancies were resolved through discussion with 2 researchers (BS and TS) who had not been involved in the initial screening process. The final database of included papers was checked again by 1 reviewer (BS) to ensure that each paper fulfilled eligibility criteria. The updated search conducted in September 2025 led to the identification of 2283 additional records. After deduplication, 1603 records remained and underwent the same screening procedure, resulting in 6 additional papers being included. This process resulted in 42 papers, with 10 additional studies identified through searching methods, totaling 52 papers. Figure 3 shows the study selection process and provides a more detailed summary of each screening stage, and Figure 4 provides an overview of the updated search.

### Intervention Characteristics

Of the 52 papers that met the inclusion criteria, 1 paper contained 2 separate experiments [48], while 2 pairs of papers reported findings from the same study conducted among the same sample [54-57]. Therefore, this review contained 51 unique studies, of which most were RCTs (n=38). The included studies were published across a wide range of disciplines, including public health, medical, communication, and psychological journals, reflecting the interdisciplinary nature of research on digital interventions and eating behavior. Most studies were conducted in developed countries, with the majority in the United States (n=10), followed by Italy (n=9), the United Kingdom (n=6), Australia (n=5), and other Western countries such as Denmark (n=2) and Belgium (n=2). Only 4 studies took place in a developing country, specifically in Mexico (n=2), China (n=1), and Saudi Arabia (n=1). All studies were published between 2004 and 2025 and were mostly conducted among adults (n=24), young adults (n=16), or adolescents (n=6). Some focused on children (n=1) or child-parent dyads (n=3), while 1 study did not disclose on population type or age [58]. Sample sizes varied considerably from 28 to 5062 participants at baseline and 24 to 1788 participants after the intervention. Altogether, the studies included 24,652 participants at baseline. More information on the study characteristics can be found in Multimedia Appendix 5 [48,50-99].

Socioeconomic characteristics were inconsistently reported, with only 56% (29/52) of the included studies providing relevant data. The most commonly used indicators were educational level (24/29, 83%), income (8/29, 28%), and occupation or working status (5/29, 17%). One study used food assistance as a proxy for income [54], and 3 incorporated area-based indicators [59-61]. Nearly all studies that included indicators were conducted among adults, and only 2 studies reported socioeconomic characteristics for adolescents. The majority demonstrated a predominance of participants with higher educational attainment, with some reporting up to 95% having tertiary education or International Standard Classification of Education levels 3 to 8. Similarly, among studies reporting income data, the proportion of participants in the lowest income category was small, ranging from 3.5% to 21%, with the exception of 1 study that reported an equal distribution between low- and middle-income groups [62]. While some studies controlled for socioeconomic indicators, few included them as moderators or created subgroups. Only 4 studies explicitly examined the role of SES in intervention effectiveness, of which 3 found significant effects on food intake among lower SES participants [56,57,62]. Additionally, Lim et al [63] found that income correlated with increased legume intake and decreased intention to consume animal-based foods. However, due to the limited and inconsistent reporting of SES as well as the underrepresentation of individuals from lower socioeconomic backgrounds, the planned moderator analysis based on SES was not feasible.

Across the 52 papers, a total of 69 digital interventions (k) were assessed. Intervention duration and intensity varied widely, spanning from 1 week to 6 months. While some were self-paced, most delivered content at intervals ranging from once a week to twice daily. The majority focused on promoting fruit and vegetable intake (FVI; 61%) as an indicator of healthy and sustainable eating behavior. A smaller proportion addressed the reduction of meat consumption (19%) or a broader healthy and sustainable diet (20%; eg, Mediterranean diet). Most interventions used SMS text messaging (41%) or websites (25%), followed by social media (13%), mobile apps (9%), games (7%), and emails (4%).

### Use of BCTs

In total, 53 unique BCTs were identified belonging to 15 of the 16 hierarchical clusters of the BCTTv1. The most frequently used BCT clusters were “goals and planning” (65%), “natural consequences” (64%), “feedback and monitoring” (52%), and “comparison of behavior” (41%). The number of BCTs per intervention ranged from 1 to 21, with an average of 6 (SD 4.5). More information on the prevalence of each BCT can be found in Multimedia Appendix 6.

### Narrative Summary of Findings by Digital Medium

#### SMS Text Messaging Interventions

Among the 28 SMS text messaging interventions, 22 (79%) showed significant effects. Most SMS text messaging interventions incorporated BCTs that aimed to inform participants about the consequences of unhealthy or unsustainable eating (BCT cluster 5; 86%). Some facilitated goal setting and planning (BCT cluster 1; 50%) or provided feedback and supported self-monitoring (BCT cluster 2; 25%). SMS text messaging interventions that draw on BCTs from clusters 5 and 2 appeared particularly promising, with significant effects in 79% and 71% of cases, respectively. Those based on cluster 1 were less consistently effective, with just over half (57%) yielding significant outcomes.

In total, 6 of 8 (75%) interventions targeting adults reported effects on eating behavior [59,64-66]. For instance, Carfora and Catellani [64] showed that a 2-week SMS text messaging intervention significantly increased legume intake and reduced meat consumption compared to a passive control group. The most effective messages leveraged dynamic norms that highlight an increase in people engaging in healthy and sustainable eating behavior. In contrast, 2 interventions aiming to improve adults’ FVI were unsuccessful [59,66]. Both studies might have had an insufficient intervention dose to foster behavior change, as participants only received messages 2 times per week or 5 times per month.

Among young adults (aged 18‐30 years), 16 SMS text messaging interventions were evaluated. Nearly all interventions (88%) demonstrated significant positive impacts on eating behavior, particularly in reducing meat intake. In total, 12 interventions decreased red or processed meat consumption, with effects lasting up to 8 weeks after the intervention [67-72]. Incorporating dynamic norms into SMS text messages about the environmental impact of meat seemed to enlarge the effects [71], which aligns with findings among adults [64]. Although most interventions successfully targeted meat reduction, those focusing on increasing intake of plant-based foods (k=4) showed mixed results [63,73,74].

A smaller subset of SMS text messaging interventions focused on adolescents (aged 14‐19 years; k=4), with 75% reporting significant effects [75,76,116]. One study reported significant differences in FVI change between intervention and control, but this was primarily due to a sharp postintervention decline in the control group rather than an increase in FVI in the IG [75]. On the contrary, Pedersen et al [76] did not find significant improvements in FVI compared to the control group, likely due to low participant engagement, as positive changes were observed only among those who sent more than 50% of the SMS text messages.

#### Web-Based Interventions

Studies assessing the effectiveness of web-based interventions (k=16) reported limited significant results [50,51,56-62,77-83]: 7 (44%) interventions showed mixed effects due to inconsistent effects across time points or eating outcomes, and 5 found no significant effects. Almost all web-based interventions included BCTs related to goal setting and planning (BCT cluster 1; 94%) and feedback and monitoring (BCT cluster 2; 81%). Other commonly used techniques targeted knowledge (BCT cluster 4; 63%), comparison of behavior (BCT cluster 6; 56%), and social support (BCT cluster 3; 50%). Incorporating feedback and monitoring was effective in 85% of cases, and 80% of the interventions targeting knowledge demonstrated effects.

Among the 14 interventions targeting adults, the most promising were the web-based self-regulation interventions. The study of Plaete et al [50] reported significant improvements in FVI, but the feasibility study only noted an effect for fruit consumption [51]. Similarly, Frie et al [52] and Stewart et al [53] examined the effects of a self-regulation intervention on meat intake, observing reductions in meat consumption 1 week after the intervention but not at 1-month follow-up. Other interventions among adults showed limited success, as they either only had significant effects during the intervention that did not persist afterward [75] or because intake increased only for specific subgroups of the sample [62,81]. In total, 4 web-based interventions did not improve adults’ eating behavior compared to the control group [56,57,60,78].

Only 2 interventions targeted the eating behavior of younger age groups. Chamberland et al [61] tested the impact of a web-based school intervention on adolescents aged 14 to 16 years, while Røed et al [80] developed a website for parents that focused on creating a healthy food environment to indirectly improve children’s FVI. Both studies showed significant postintervention effects on FVI, but the effects did not persist at 3- to 6-month follow-up.

#### Social Media Interventions

In total, 9 interventions tested the effectiveness of a social media intervention, with 5 reporting significant effects. The most frequently incorporated BCT clusters were goals and planning (BCT cluster 1; 67%), social support (BCT cluster 3; 44%), natural consequences (BCT cluster 5; 56%), and comparison of behavior (BCT cluster 6; 56%). BCT clusters 3 and 6 appear most promising in social media intervention, as 75% and 80% of the interventions using them demonstrated significant effects.

In total, 4 interventions were conducted among young adults, 4 among adults, and 1 did not report the target group of the intervention. In 1 study of Kilb et al [48], young adults participated as dyads in an intervention, in which senders were asked to post about fruit and vegetables on Facebook, and network members were exposed to these messages. Neither senders nor network members significantly increased their FVI compared to control dyads. In contrast, the second study of this paper found that both private and public self-monitoring via social media increased FVI [48]. Similarly, Meng et al [84] showed that group-based self-tracking on a (researcher-developed) social network website led to a greater increase in FVI compared with individual self-tracking. A recent study of Hawkins et al [85] highlighted that mere exposure to healthy food content on Instagram can improve young adults’ FVI.

Among adults, an intervention with support groups reported a significant increase in FVI during the intervention, but these effects were not maintained at follow-up [86]. In Ng et al [87], participants’ FVI increased after completing a 4-week intervention containing recipes and videos delivered via Facebook. Carreño Enciso et al [88] tested an educational intervention delivered via Instagram or Facebook but found no significant effects on adherence to the Mediterranean diet. Weber and Nigg [58] tested an intervention containing motivational YouTube videos related to healthy eating. No changes were observed in FVI, which may be due to the low intervention dose (ie, only 6 exposures) and the requirement for participants to actively expose themselves to the intervention.

#### Mobile App Interventions

Mobile apps were used in 7 interventions. All incorporated feedback and monitoring techniques (BCT cluster 2), while 5 interventions also applied BCTs related to goals and planning (BCT cluster 1), and 4 addressed the consequences of unhealthy eating (BCT cluster 5). However, the effectiveness of these techniques within mobile app interventions appears limited, as only 40%‐57% of the interventions incorporating these clusters reported significant effects.

In total, 3 of 7 app-based interventions reported significant effects. Hendrie et al [89] tested an app that included different sections with recipes and feedback in a real-world setting and found an increase in vegetable intake. The PersuHabit app of Vázquez-Paz et al [90] effectively targeted parents in order to promote FVI among young children, and Liu et al [91] found that a food evaluation app significantly improved the animal-to-plant food ratio in employees’ lunches. These studies were conducted among adults, while the 4 interventions showing no or mixed results targeted (pre or late) adolescents (aged 9‐18 years) [92-94].

#### Interventions Using Games or Emails

While apps appear to be more effective among adults, games tend to yield better results in child populations. The FoodRateMaster game of Espinosa-Curiel et al [95] significantly increased FVI intake among children. Thompson et al [96] targeted both parents and children to improve children’s FVI and found that games were effective in improving children’s diets, but only when they contained action planning. One study tested an intervention featuring 3 games among adults but found no improvement in their FVI [97]. However, it is important to note that this was one of the oldest studies included in this review; therefore, the gaming experience may have differed from those of more recent studies. Since 6 BCT clusters were applied in over 80% of the game-based interventions, it is difficult to explore the most used and promising BCTs in gaming interventions.

A limited number of studies tested email interventions (k=3). Rompotis et al [74] found that habit-based messages providing strategies to strengthen the automaticity of FVI were more effective in improving young adults’ fruit intake compared to general nutrition information, regardless of whether the messages were sent via texting or email. Block et al [98] reported similar results for a worksite email intervention among adults. One study did not find significant effects on young adults’ FVI [99], which could be explained by the limited intervention dose (30 days with emails every 3 days), compared to the 2 other interventions, which lasted 8 to 12 weeks [74,98]. All of the emailing interventions included BCTs related to goals and planning (BCT cluster 1).

### Effectiveness of Digital Interventions

#### Overview

**Figure 5.**
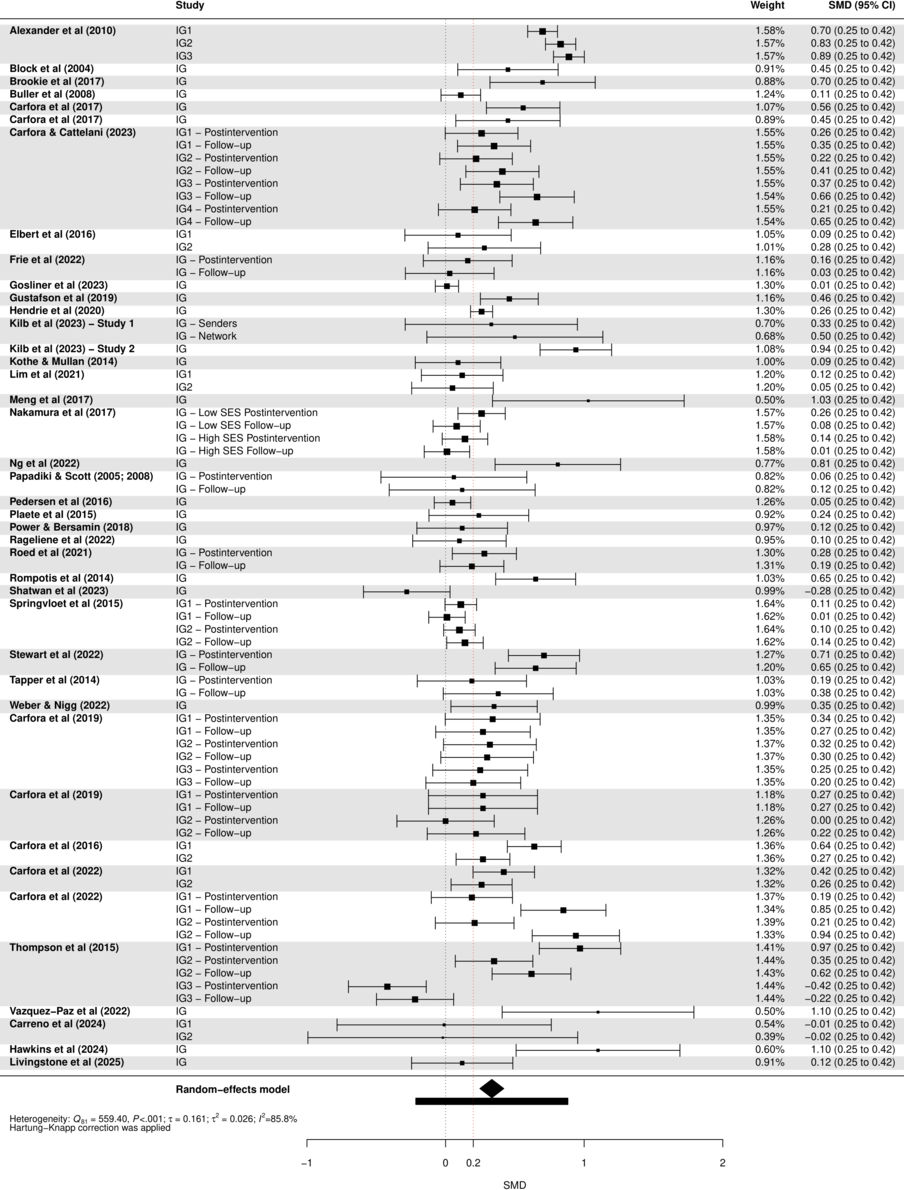
Forest plot of standardized mean differences (Cohen *d*) for the effect of digital interventions on healthy and sustainable eating (ie, increased plant-based intake or reduced meat intake; for study details, see Multimedia Appendix 5) [48,51-56,58-60,62-71,73-77,79,80,82,84,85,87-90,92-94,96,98,99,116]. IG: intervention group; PI: prediction interval; SES: socioeconomic status; SMD: standardized mean difference.

**Figure 6.**
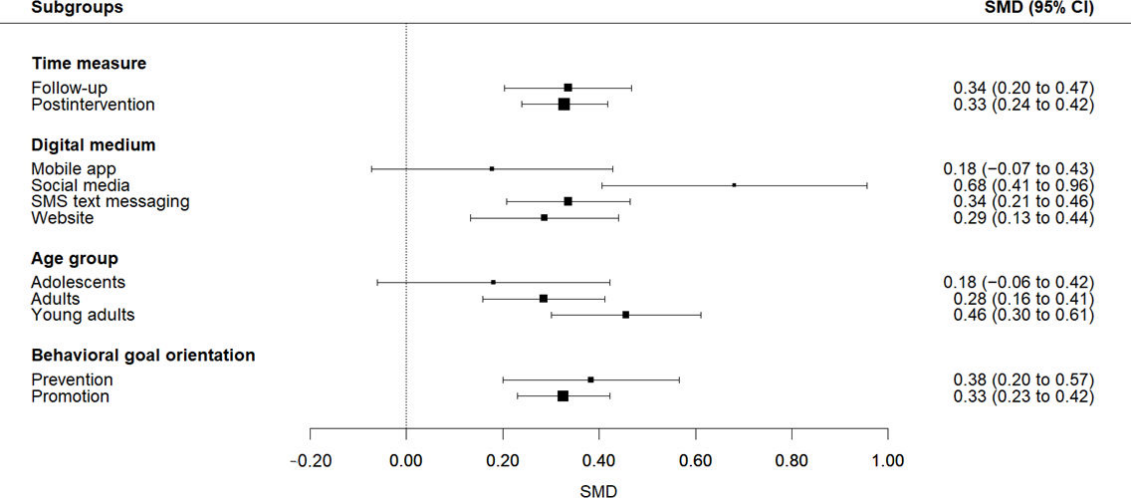
Forest plot of the pooled standardized mean differences (Cohen *d*) for each subgroup of the moderator analyses. SMD: standardized mean difference.

Of the 52 included studies, 41 provided sufficient data for inclusion in the meta-analysis. The results show a pooled effect size of 0.33 (95% CI 0.25‐0.42; *P*<.001), indicating that digital interventions on average have a small, positive effect on healthy and sustainable eating behavior. The forest plot in Figure 5 presents the effect size for each intervention with its 95% CI. Outlier analysis identified 19 potential outliers; however, these were fairly uniformly distributed. Both the graphical representation and sensitivity analysis gave no clear indication of bias (Multimedia Appendix 7). Visual inspection of the funnel plot for small-study effects suggested limited asymmetry (Multimedia Appendix 7), which was supported by the nonsignificant value of the Egger test (*P*=.18). After removing high risk-of-bias studies, the analysis yielded similar results (*d*=0.36, 95% CI 0.27‐0.45; *P*<.001). The sensitivity analysis excluding 8 effect sizes from NRS yielded results highly consistent with the primary analysis (*d*=0.32, 95% CI 0.23‐0.41; *P*<.001), indicating that the findings are robust to study design. However, heterogeneity statistics revealed substantial heterogeneity (*Q*_81_=559.40; *P*<.001; *τ*=0.16; *τ^2^*=0.03; *I*^2^=86%), which was supported by the 95% PI, which ranged from −0.21 to 0.87. Given the substantial heterogeneity, we cannot be confident that the positive effect is robust. True effects may vary considerably in settings, with both negative effects as well as strong positive effects being possible. Moderator analyses with categorical variables (ie, subgroup analyses) were conducted to explore between-study variance and provide a more nuanced understanding of the effectiveness of digital interventions. Forest plots for the subgroup analyses can be found in Figure 6.

#### Postintervention and Follow-Up Effects

A meta-analysis with time as covariate yielded similar effect sizes for outcomes measured during or immediately after the intervention (*d*=0.33, 95% CI 0.24‐0.42; *P*<.001; *I*^2^=65%, 95% PI −0.17 to 0.85) and outcomes measured 1 to 6 months after the intervention (*d*=0.34, 95% CI 0.20‐0.47; *P*<.001; *I*^2^=77%, 95% PI −0.29 to 0.84). The test for subgroup differences shows that there are no significant differences between postintervention and follow-up effects (*Q_M_*=0.01, *df*=1; *P*=.91). These results held when analyses were restricted to RCTs only (Multimedia Appendix 7). This suggests that, on average, the effectiveness of the digital interventions was quite stable over time, with no meaningful difference in effect between the postintervention and follow-up measures.

#### Behavioral Goal Orientation

The analysis with behavioral goal orientation as a moderator assessed whether promotion-focused and prevention-focused interventions differed significantly in effectiveness. Digital interventions with a promotion focus (k=47), aimed at encouraging intake of plant-based foods, yielded a statistically significant, small pooled effect size of *d*=0.33 (95% CI 0.23‐0.42; *P<.*001). Heterogeneity was still substantial in this subgroup (*I*^2^=70%, 95% PI −0.23 to 0.88). Prevention-focused interventions (k=12), all of which targeted reductions in red or processed meat consumption, demonstrated a slightly larger pooled effect size of *d=*0.38 (95% CI 0.20‐0.57; *P*<.001) and lower heterogeneity (*I*^2^=64%, 95% PI −0.08 to 0.85). The effect difference between promotion and prevention studies was not significant (*Q_M_*=0.29, *df*=1; *P*=.59). The sensitivity analysis restricted to RCTs yielded comparable findings (Multimedia Appendix 7).

#### Age Group

Including participant age group as a moderator in the meta-analysis revealed that digital interventions had the largest effects among young adults (Table 1). In contrast, the smallest pooled effect size was found among adolescents; however, this finding is based on only 6 interventions. The test of moderators did not indicate a significant difference in pooled effect sizes across age groups (*Q_M_*=4.51, *df*=2; *P*=.10). Effect estimates remained consistent when the analysis was restricted to RCTs (Multimedia Appendix 7).

**Table 1.** Meta-analysis for the effect of digital interventions with age group as moderator.

Age group	*k*	*d*	95% CI	*I*^2^ (%)	95% PI^a^
Adults	26	0.28^b^	0.16 to 0.41	64	−0.19 to 0.75
Young adults	19	0.46^b^	0.30 to 0.61	65	−0.10 to 1.00
Adolescents	6	0.18	−0.06 to 0.42	86	−0.45 to 0.84

a PI: prediction interval.

b *P*<.001.

#### Digital Medium

The analysis with type of digital medium as moderator was significant (*Q_M_*=8.49, *df*=3; *P*=.03), indicating that the mode of delivery modified the pooled effect. Interventions delivered via social media or SMS text messages yielded the largest effect sizes (Table 2). Despite the low number of social media interventions, pairwise comparison showed a significant subgroup difference with higher effectiveness for social media than for SMS text messaging (*z*=2.23; *P*=.03), website (*z*=2.56; *P*=.01), and mobile app interventions (*z*=2.64; *P*=.01). The heterogeneity score for social media interventions was moderate, and the positive PI suggests that future social media interventions are likely to yield positive effects, although the lower bound is close to 0 (*I*^2^=47%, 95% PI 0.01‐1.31). Exclusion of NRS did not meaningfully alter the results, except that the subgroup difference between social media and SMS text messaging interventions was no longer statistically significant (*z*=1.72; *P*=.09). Additionally, the sensitivity analysis for mobile app interventions could not be conducted due to an insufficient number of studies (Multimedia Appendix 7).

**Table 2.** Meta-analysis for the effect of digital interventions with digital medium as moderator.

Digital medium	*k*	*d*	95% CI	*I*^2^ (%)	95% PI^a^
SMS text messaging	24	0.34^b^	0.21 to 0.47	62	−0.05 to 0.73
Website	13	0.28^b^	0.13 to 0.43	77	−0.25 to 0.80
Social media	8	0.65^b^	0.41 to 0.90	47	0.01 to 1.31
Mobile app	5	0.18	−0.07 to 0.43	72	−0.53 to 0.93

a PI: prediction interval.

b *P*<.001.

#### BCT Clusters

Meta-analyses with the presence of each BCT cluster found the largest standardized mean difference for interventions incorporating prompts or cues (BCT cluster 7), with a pooled effect size of *d*=0.62 (95% CI 0.33‐0.91; *P*<.001). The test of moderators indicated that interventions including this BCT cluster were significantly associated with a greater improvement in eating behavior than those without this cluster (*P*=.04). Interventions including social support (cluster 3) and comparison of behavior (cluster 6) demonstrated higher pooled effect sizes (*d*=0.39) compared to those that did not include these BCTs (*d*=0.31 and *d*=0.29, respectively). Although these differences were not statistically significant (*P*=.40 and *P*=.17, respectively), the numerically higher effect sizes suggest that these BCTs may still contribute positively to intervention outcomes. The lack of significance could be attributed to the limited number of studies incorporating these BCT clusters, underscoring the need for further research.

Several BCT clusters showed similar effect sizes regardless of whether they were present in interventions or not. For instance, feedback and monitoring (cluster 2), repetition and substitution (cluster 8), and antecedents (cluster 12) had comparable pooled effect sizes in both subgroups, suggesting no clear added value of including these BCTs (Table 3). Interventions that incorporated the BCT clusters “shaping knowledge” (cluster 4) and “natural consequences” (cluster 5) showed smaller pooled effect sizes compared to those in which these clusters were absent; however, these differences were not statistically significant (*P*=.07 and *P*=.35, respectively). Overall, restricting these moderator analyses to RCTs produced similar results (Multimedia Appendix 7). Moderator analyses were also conducted for individual BCTs, which generated similar findings (Multimedia Appendix 3).

**Table 3.** Meta-analysis for the effect of digital interventions with behavior change technique (BCT) cluster as moderator.

BCT cluster	BCT cluster present			BCT cluster absent			Test of moderators
	*k*	*d*	95% CI	*k*	*d*	95% CI	*P* value
1. Goals and planning	37	0.31^a^	0.19 to 0.43	20	0.37^a^	0.24 to 0.49	.56
2. Feedback and monitoring	32	0.33^a^	0.20 to 0.47	25	0.33^a^	0.22 to 0.44	>.99
3. Social support	16	0.39^a^	0.21 to 0.56	41	0.31^a^	0.22 to 0.40	.40
4. Shaping knowledge	27	0.25^b^	0.10 to 0.39	30	0.40^a^	0.29 to 0.52	.07
5. Natural consequences	39	0.30^a^	0.22 to 0.39	18	0.39^b^	0.18 to 0.59	.35
6. Comparison of behavior	27	0.39^a^	0.27 to 0.51	30	0.29^a^	0.18 to 0.39	.17
7. Associations	6	0.58^a^	−0.10 to 1.25	51	0.30^a^	0.23 to 0.38	.04
8. Repetition and substitution	12	0.30^b^	0.15 to 0.45	45	0.34^a^	0.24 to 0.44	.64
9. Comparison of outcomes	9	0.38	−0.06 to 0.83	48	0.32^a^	0.24 to 0.41	.60
10. Reward and threat	13	0.36^b^	0.13 to 0.60	44	0.32^a^	0.23 to 0.42	.72
12. Antecedents	6	0.33^b^	0.11 to 0.55	51	0.33^a^	0.24 to 0.42	.97
13. Identity	10	0.19^c^	0.07 to 0.30	47	0.36^a^	0.27 to 0.46	.09
14. Scheduled consequences	4	0.29	−0.74 to 1.32	53	0.34^a^	0.25 to 0.42	.78
15. Self-belief	7	0.29	−0.09 to 0.67	50	0.34^a^	0.24 to 0.43	.75

a *P*<.001.

b *P*<.01.

c *P*<.05.

## Discussion

### Principal Findings

While the meta-analysis found a moderate and statistically significant overall effect of digital interventions on healthy and sustainable food consumption, the substantial heterogeneity observed across studies suggests that these interventions do not have a consistent effect across all populations and contexts. Additionally, included studies mostly had a moderate risk of bias, and together with the inconsistency in effects, this may reduce the certainty of the evidence regarding the overall effectiveness of digital interventions. Both the narrative review and the moderator analyses showed that the effectiveness of digital interventions varied by multiple characteristics and settings.

Regarding the food outcomes, interventions preventing animal-based food consumption and those promoting plant-based food intake both yielded a significant, moderate effect. While interventions with a prevention focus yielded a slightly larger pooled effect size, moderator analyses showed no significant difference with interventions promoting plant-based food intake. This contrasts with prior research, suggesting that a promotion focus is more effective in encouraging healthy dietary patterns [26,117]. However, this might be explained by a difference in the food consumption studied. For example, the studies reviewed by Zlatevska et al [117] primarily targeted discretionary items such as sugary drinks and snacks, not meat consumption. Only 1 of the included interventions in this review combined a prevention and promotion strategy and found sustained effects at follow-up [64], highlighting a need for future research to test more integrative approaches that align closely to the EAT-Lancet diet.

Both the narrative synthesis and moderator analyses show that the effectiveness of digital interventions varies by modality. SMS text messaging interventions had a moderate effect on healthy and sustainable food intake, with over 75% demonstrating significant results in the narrative review. Despite the small number of social media interventions, they had the strongest effects on eating behavior, being significantly associated with larger effect sizes than other modes of delivery. While prior research associates social media use with unhealthy eating habits [118,119], these findings suggest that social media can also be used to positively influence dietary behavior. Since many consumers engage with food content on social media, platforms such as Instagram or Facebook hold great potential to promote healthier and more sustainable eating [120,121]. Social media platforms present unique environments in which users can form large social networks, allowing them to seek information about others’ behavior and receive positive reinforcements for their own behavior. Although the evidence base remains limited, social media interventions may promote stronger behavior change for several reasons: they expose users to peers’ behaviors (social modeling), leverage participants’ existing engagement with the platform to sustain intervention exposure and retention, and offer interactive features that facilitate social support [122,123]. Notably, none of the social media interventions targeted adolescents, despite this being a key demographic due to their high engagement with social media and high exposure to unhealthy food marketing [124,125].

These findings suggest that interventions delivered through accessible and familiar platforms (ie, SMS text messaging or social media) tend to be more effective than interventions requiring more intentional user engagement (ie, stand-alone websites or apps). This difference also links to the amount of agentic demand (ie, the degree to which participants are required to engage with the content in order to achieve the intended outcome) [126,127], though this requires further empirical testing. The common academic practice of developing digital interventions via research-created platforms, often used only briefly and actively throughout the study period, implies a misalignment with real-world digital behavior [35]. To enhance ecological validity and long-term behavior change, future studies could focus on accessible interventions using existing, familiar platforms [126,128]. While social media interventions were significantly more effective than interventions via other platforms, they also present specific challenges for researchers. As opposed to more controlled intervention environments such as apps or websites, social media constitute open and dynamic spaces that are saturated with unhealthy food marketing and health misinformation [129]. This may dilute or even counteract intervention effects and can lead to nutrition confusion [130].

A wide range of BCTs was incorporated across the interventions, and the choice of BCT varied depending on the type of digital medium used. BCTs targeting goals and planning were most frequently applied across all interventions, particularly in web-based interventions. Knowledge-related techniques, such as providing instructions, were frequently applied, especially in web-based interventions or games. Communicating the consequences of unhealthy eating behavior was also a commonly used cluster of techniques, mainly in SMS text messaging interventions. Yet, these information-based BCTs were not the most effective ones. Although definitive conclusions on the most effective BCT cluster cannot be made due to the limited number of studies incorporating certain clusters, moderator analyses [1] indicated that interventions incorporating prompts or cues (BCT cluster 7) were significantly more effective than those that did not, yielding a large effect size. Additionally, interventions targeting social mechanisms through social support (cluster 3) or comparison of behavior (cluster 6) demonstrated larger effects, though not significantly larger than those not including these BCTs. The narrative synthesis further suggests that social norm communication is an effective strategy, as several studies with significant effects incorporated descriptive norms to influence behavior. These findings suggest that future interventions may benefit from shifting emphasis away from purely informational strategies (eg, raising awareness or increasing knowledge) and instead testing techniques that leverage social influence, peer dynamics, and cues. Previous research on health interventions also suggests that providing social support strengthens the impact of digital interventions [131-133], and that norm communication can gradually reshape individuals’ perceptions of others’ behavior, prompting them to align with these evolving norms [134-136].

Finally, while age-related differences in the effect size of the interventions were not significant, moderator analyses yielded the strongest effect size for young adults. Due to the limited number of studies targeting adolescents, conclusions for this age group cannot be drawn. Regarding SES, most studies included SES indicators only as control variables rather than focusing on targeted recruitment, resulting in the underrepresentation of lower SES individuals. This limited our ability to conduct subgroup analyses for this population. This lack of research is concerning, given that food intake is socially structured, with unhealthy and unsustainable diets being more prevalent among lower SES individuals [137-139]. While financial constraints and educational disparities contribute to these patterns, modifiable psychosocial factors (eg, attitudes, literacy, desired identity, or social norms) also play a critical role [140-142]. Despite potential challenges related to digital access and literacy, it is crucial to reach lower SES populations in the current digital era to help mitigate health inequalities related to dietary behavior, especially adolescents, given that they are highly active on digital platforms. Leveraging platforms such as social media may help embed interventions within their daily routines and reduce barriers to participation. Future research should conduct both targeted testing of interventions within lower SES groups and exploration of SES moderation effects to understand intervention effectiveness across socioeconomic groups.

This review reveals some major gaps in the literature. First, individuals from lower socioeconomic backgrounds remain underrepresented in research testing digital interventions. None of the included studies used subjective measures of SES (ie, perceptions of own social status) [2] despite evidence that perceived SES is a strong predictor of health (behavior) even after controlling for objective indicators [143-145]. Future research should therefore not only systematically measure SES but also incorporate subjective indicators more frequently [146]. Second, while the included interventions targeted various age groups, few studies specifically focused on adolescents. The handful of studies that included adolescents as participant group primarily tested web-based or mobile app interventions, which were among the least effective digital modalities. Both the lack of research as well as the prominent focus on websites or apps may explain the smaller effect size for this demographic. Future research should prioritize low-agentic interventions for adolescents of different socioeconomic backgrounds, particularly through social media, as adolescents not only increasingly use these platforms, but research also shows that exposure to social media food content influences their eating behavior [118,124]. Finally, most studies focused on specific food groups, particularly fruits and vegetables, framing them as key components of a healthy diet. These findings are similar to previous systematic reviews on interventions aiming to improve food consumption [33,147]. While fruits and vegetables are indeed a critical part of a healthy and sustainable diet, interventions should also highlight other important components, such as legumes and whole grain products. Furthermore, rather than solely focusing on reducing meat consumption, interventions can promote replacing meat with whole-food plant-based alternatives, as substituting certain products is often more achievable than complete elimination [18,64].

### Implications

By identifying the conditions in which digital interventions are most effective for encouraging plant-based eating, this study offers timely guidance for practitioners in a rapidly evolving digital landscape. The results of this review suggest that digital interventions can effectively be implemented to support people in transitioning to healthier and more sustainable eating behavior. Given the variability in effectiveness following intervention characteristics, health care providers and practitioners should consider selecting digital platforms that best fit the preferences, literacy, and lifestyle of the target group. Social media may be particularly useful for delivering low-threshold interventions in community settings, targeting mechanisms of social influence for behavior change. Practitioners are encouraged to adopt evidence-based BCTs that leverage social influence (ie, social support and demonstration of behavior) and thus shift emphasis from purely informational strategies (eg, raising awareness or increasing knowledge). Collaboration between behavioral scientists and health care professionals can help ensure that interventions are evidence-based and aligned with participants’ needs. Special attention is needed for the participation of underrepresented groups, more specifically, adolescents and individuals with lower SES. Future research should use specific strategies to recruit and engage these vulnerable populations, for example, through school-based programs or partnerships with community organizations. Moreover, it is important to ensure that intervention materials are accessible and inclusive to help reduce disparities in participation and retention. One way to ensure this is by actively involving members of the vulnerable group or trusted intermediaries in the design of the intervention [148].

### Limitations

Despite using extensive search strategies, relevant research may have been overlooked if published in languages other than English or if they were inaccessible through the selected databases. The majority of included studies demonstrated moderate risk of bias, with measurement of outcome bias being the most prevalent concern due to reliance on self-reported dietary measures rather than objective assessments. This may lead to overestimation of intervention effects, as self-report measures are susceptible to social desirability bias and recall error. Future research should prioritize objective outcome measures to strengthen evidence for real-world applicability. In total, 11 studies could not be included in the meta-analysis due to insufficient data for effect size calculation. Additionally, the inconsistency in measuring SES and the scarcity of research involving participants from lower SES backgrounds hindered our ability to assess the differential effects of digital interventions across SES groups. Moreover, the small number of studies contributing data to certain moderators may have limited the statistical power to detect potential moderation effects [149] and restricted our ability to assess their combined influence. Ideally, a sufficiently large number of studies would be available to allow the modeling of joint effects, rather than examining each moderator separately. Furthermore, most evidence was derived from studies with a moderate risk of bias, and substantial heterogeneity was present. Although the subgroup analyses explained some of the observed variation, these factors may still affect the overall certainty of the evidence. We did not perform a formal Grading Recommendations Assessment, Development, and Evaluation assessment to systematically rate the certainty of the evidence, which limits our ability to evaluate the confidence in our findings [150].

Another potential limitation is the inclusion of both RCTs and NRS in the meta-analysis. The appropriateness of combining different study designs remains debated in the methodological literature, with studies having inconsistent conclusions about the risks of pooling RCTs and NRS (eg, achieving statistical significance only after inclusion of NRS) [109,151,152]. We retained NRS in our primary analyses for 2 reasons. First, sensitivity analyses excluding NRS demonstrated that our findings were robust, with effect estimates, CIs, and heterogeneity statistics remaining consistent. Second, NRS are increasingly recognized as valuable sources of evidence, also within digital intervention research, as ecological validity and real-world application are important criteria. In this context, NRS can provide complementary evidence alongside RCTs [153,154]. However, the pooled estimates should therefore be interpreted with awareness of some methodological considerations. RCTs and NRS are susceptible to different types of bias [151,152]. RCTs face risks related to randomization, allocation concealment, and blinding, while NRS are prone to confounding and selection bias, which is reflected in the different risk-of-bias assessment tools used for each design. The pooled estimates, therefore, represent weighted averages that incorporate these different types of bias, which may complicate direct translation of the results to practice [151,152]. Moreover, these study designs provide different types of evidence: RCTs answer questions of effectiveness under controlled conditions, while NRS can, for instance, address generalizability or real-world performance [151,153]. Consequently, the primary findings should be interpreted as reflecting the overall evidence base for digital interventions, rather than as a pure estimate of intervention effectiveness derived solely from controlled trials. Importantly, the consistency of the findings in the sensitivity analyses restricted to RCTs supports the robustness of the study’s main conclusions regarding intervention effectiveness.

Other important limitations are with regard to the BCTs. Categorizing intervention components into BCTs based on study descriptions and protocols proved challenging, as studies used varying terminology and levels of detail on the intervention content. Similar issues have been highlighted in previous systematic reviews [36,146]. Therefore, despite the use of a coding manual and following a training in BCT taxonomy, the presence of some techniques may have been overlooked. To enhance the replicability of interventions and accuracy of coding BCT presence for meta-analyses, future research should describe intervention content in greater detail and more systematically by following taxonomies or reporting guidelines [24,155]. It is also essential to recognize that BCTs are not exhaustive; they possess a certain level of superficiality. While BCTs can be implemented in various ways, the content was generalized to a certain type of BCT, potentially masking variations in their implementation. For instance, informing participants about the components of a healthy diet or providing personalized recipes both fall under the same BCT category (4.1 Instruction on how to perform the behavior) but represent distinct approaches that may yield different effects. Moreover, some characteristics of persuasive communication are overlooked by focusing on BCTs (eg, framing of information).

### Conclusions

This review provides a comprehensive overview of digital interventions, suggesting that digital interventions can effectively improve eating behavior, though their success varies by intervention design and population targeted. Social media emerge as particularly promising, likely due to their unique social and interactive features. Importantly, the evidence base mainly consists of studies with a moderate risk of bias, highlighting the need for more high-quality studies to confirm current results. Moreover, the meta-analytic results have a broad PI, indicating that while the average effect is positive, individual interventions may range from highly effective to potentially ineffective, depending on context and design. To our knowledge, it is one of the first reviews to systematically code the characteristics of digital interventions, including their mode of delivery (ie, digital medium), content (ie, BCTs), behavioral goal orientation (prevention vs promotion), and targeted demographic (ie, age and SES), and to link these with the intervention effect size. Despite our aim to explore effects specifically among low SES groups, the limited available research restricted our ability to conduct subgroup analyses for this population. Our findings offer valuable insights for practitioners and researchers interested in leveraging digital media for behavior change by providing an evidence base on the contexts and types of digital interventions that most effectively promote plant-based eating.

## Supplementary material

10.2196/80821 Multimedia Appendix 1 Search string.

10.2196/80821 Multimedia Appendix 2 Risk of bias.

10.2196/80821 Multimedia Appendix 3 Moderator analyses—behavior change techniques.

10.2196/80821 Multimedia Appendix 4 Calculation of effect sizes and meta-analysis.

10.2196/80821 Multimedia Appendix 5 Study characteristics.

10.2196/80821 Multimedia Appendix 6 Prevalence of behavior change techniques.

10.2196/80821 Multimedia Appendix 7 Sensitivity analyses.

10.2196/80821 Checklist 1 PRISMA and PRISMA-S checklist.

## Abbreviations

BCT: behavior change technique
BCTTv1: Behavior Change Technique Taxonomy version 1
FVI: fruit and vegetable intake
IG: intervention group
NRS: nonrandomized study
PI: prediction interval
PRISMA: Preferred Reporting Items for Systematic Reviews and Meta-Analyses
PRISMA-S: Preferred Reporting Items for Systematic Reviews and Meta-Analyses Literature Search Extension
RCT: randomized controlled trial
SES: socioeconomic status
