# Supplementary material for: Digital Interventions Targeting Healthy and Sustainable Eating Behavior: Systematic Review and Meta-Analysis
Source: J Med Internet Res. 2026 Jan 8;28:e80821. doi: 10.2196/80821 (PMC12782463; doi:10.2196/80821)
Supplement: Multimedia Appendix 1 [file jmir-v28-e80821-s001.docx]

Web of Science

**(TS**=(human OR consumer OR child* OR adolescen* OR adult OR senior OR people OR person OR teen* OR “young adult” OR girl OR boy OR youth OR student OR wom?n OR men OR man)

**AND TS**=((digital* OR app OR application OR mobile OR web OR “web-based” OR online OR internet OR computer* OR e$health OR m$health OR “text messag*” OR sms OR email OR “e-mail” OR "social medi*" OR “social network*” OR "social networking site" OR SNS OR Instagram OR Facebook OR Snapchat OR TikTok TikTok OR LinkedIn OR Messenger OR Pinterest OR Twitter OR MySpace OR Youtube OR Whatsapp OR Bereal OR Reddit OR WeChat OR Douyin OR QQ OR Weibo OR Kuaishou OR Qzone OR Telegram OR Tieba OR Quora OR Viber OR Xiaohongshu OR tweet OR “online communit*” OR “online platform*” OR “social platform*”) NEAR/10 (*intervention* OR *campaign* OR *promotion* OR *program*))

**AND TS** =(((health* OR core* OR energ* OR nutrient-rich OR “low-fat”) NEAR/5 (nutri* OR food OR eat* OR meal OR cook* OR beverage OR drink* OR diet* OR nourishment OR consum* OR product)) OR ((sustainab* OR “fair trade” OR “green” OR ecological* OR “climate friend*” OR “environmentally friendl*” OR “eco$friendl*” OR “environmentally conscious” OR organic OR “plant-based” OR vegetarian* OR vegan* OR flexitarian*) NEAR/5 (nutri* OR food OR eat* OR meal OR cook* OR beverage OR drink* OR diet* OR nourishment OR consum* OR product OR diary OR meat)) OR ((avoid* OR less OR reduc* OR substitut* OR alternative OR replace* OR moderate) NEAR/5 (“meat” OR animal-based)) OR (vegetable OR fruit OR “whole grain” OR vegan OR vegetarian OR flexitarian) )

**AND TS**=(consum* OR intake OR behavio$r OR choice OR purchas* OR buy* OR eat* OR habit OR prefer* OR intent* OR choos* OR select* OR diet* OR deci*)

**NOT TI**=(alcohol* OR drug*))

**AND** (DT==("ARTICLE") **AND** LA==("ENGLISH"))

Scopus

( ( ALL(human OR consumer OR child* OR adolescen* OR adult OR senior OR people OR person OR teen* OR "young adult" OR girl OR boy OR youth OR student OR woman OR women OR man OR men ) )

AND ( TITLE-ABS-KEY ( ( digital* OR app OR application OR mobile OR web OR online OR internet OR computer* OR ehealth OR mhealth OR “e-health” OR “m-health” OR "social medi*" OR "social networking site*" OR sns OR Instagram OR Facebook OR Snapchat OR TikTok OR LinkedIn OR Messenger OR Pinterest OR Twitter OR MySpace OR Youtube OR Whatsapp OR BeReal OR Reddit OR WeChat OR Douyin OR QQ OR Weibo OR Kuaishou OR Qzone OR Telegram OR Tieba OR Quora OR Viber OR Xiaohongshu OR tweet OR OR “online communit*” OR “online platform*” OR "social platform*")
W/10 ( intervention* OR campaign* OR promotion* OR program* ) ) )

AND ( TITLE-ABS-KEY ( ( ( health* OR core OR nutrient-rich OR "low-fat" ) W/5 ( nutri* OR food OR eat* OR meal OR cook* OR beverage OR drink* OR diet* OR nourishment OR consum* OR product ) )

OR ( ( sustainab* OR "fair trade" OR "green" OR ecological* OR "climate friendl*" OR "environmentally friendl*" OR "eco-friendly" OR ecofriendly OR "eco friendly" OR "environmentally conscious*" OR organic OR "plant-based" OR vegetarian OR vegan OR flexitarian ) W/5 ( nutri* OR food OR eat* OR meal OR cook* OR beverage OR drink* OR diet* OR nourishment OR consum* OR product OR diary OR meat) )

OR ( ( avoid* OR less OR reduc* OR substitut* OR alternative OR replace* OR moderate) W/5 ( meat OR “animal-based” OR “animal source” OR “animal-source”) )

OR ( vegetable OR fruit OR "whole grain" OR vegan OR vegetarian OR flexitarian) ) )

AND ( TITLE-ABS-KEY ( consum* OR intake OR behav* OR choice OR purchas* OR buy* OR eat* OR habit OR prefer* OR intent* OR choos* OR select* OR diet* OR deci* ) ) )

AND NOT TITLE ( alcohol* OR drug* )

AND ( LIMIT-TO ( DOCTYPE,"ar" ) ) AND ( LIMIT-TO ( LANGUAGE,"English" ) )

EMBASE

**#15**

#14 AND 'article'/it AND [english]/lim

**-----**

**#14**

#3 AND #4 AND #9 AND #12 NOT (alcohol*:ti OR drug*:ti)

**----**

**#13**

#3 AND #4 AND #9 AND #12

**-----**

**#12**

#10 OR #11

**-----**

**#11**

consum*:ti,ab,kw OR intake:ti,ab,kw OR behav*:ti,ab,kw OR choice:ti,ab,kw OR purchas*:ti,ab,kw OR buy*:ti,ab,kw OR eat*:ti,ab,kw OR habit*:ti,ab,kw OR prefer*:ti,ab,kw OR intent*:ti,ab,kw OR choos*:ti,ab,kw OR select*:ti,ab,kw OR diet*:ti,ab,kw OR deci*:ti,ab,kw

**-----**

**#10**

'feeding behavior'/exp OR 'food intake'/exp OR 'nutrition'/exp

**-----**

**#9**

**#5 OR #6 OR #7 OR #8**

**----**

**#8**

vegetable*:ti,ab,kw OR fruit*:ti,ab,kw OR 'whole grain*':ti,ab,kw OR vegan:ti,ab,kw OR vegetarian:ti,ab,kw OR flexitarian:ti,ab,kw

**----**

**#7**

((sustainab* OR 'fair trade' OR green OR ecological* OR 'climate friendl*' OR 'environmentally friendl*' OR 'eco friendl*' OR 'eco-friendl*' OR 'ecofriendl*' OR 'environmentally conscious' OR organic OR 'plant-based' OR vegetarian OR vegan OR flexitarian) NEAR/5 (nutri* OR food* OR eat* OR meal OR cook* OR beverage* OR drink* OR diet* OR nourishment OR consum* OR product* OR diary OR meat)):ti,ab,kw

**----**

**#6**

((avoid* OR less OR reduc* OR substitut* OR alternative OR replace* OR moderate) NEAR/5 (meat OR 'animal-based' OR 'animal source' OR 'animal-source')):ti,ab,kw

**----**

**#5**

((health* OR core OR 'nutrient-rich' OR 'low-fat') NEAR/5 (nutri* OR food* OR eat* OR meal* OR cook* OR beverage* OR drink* OR diet* OR nourishment* OR consum* OR product)):ti,ab,kw

**----**

**#4**

((digital* OR app OR application* OR mobile OR web OR 'web-based*' OR online OR internet OR phone* OR computer* OR ehealth OR 'e-health' OR mhealth OR 'm-health' OR 'text messag*' OR sms OR email OR 'e-mail' OR 'social medi*' OR 'social networking site*' OR sns OR instagram OR facebook OR snapchat OR tiktok OR linkedin OR messenger OR pinterest OR twitter OR myspace OR youtube OR whatsapp OR bereal OR reddit OR wechat OR douyin OR qq OR weibo OR kuaishou OR qzone OR telegram OR tieba OR quora OR viber OR xiaohongshu OR tweet OR ‘online communit*’ OR ‘online platform*’ OR 'social platform*') NEAR/10 (intervention* OR campaign* OR promotion* OR program*)):ti,ab,kw

**----**

**#3**

#1 OR #2

**----**

**#2**

human OR consumer OR child* OR adolescen* OR adult* OR senior OR people OR person* OR teen* OR 'young adult*' OR girl* OR boy* OR youth OR student* OR parent* OR woman OR women OR man OR men

----

**#1**

'human'/exp OR 'consumer'/exp OR 'adolescence'/exp OR 'adolescent'/exp OR 'adult'/exp OR 'child'/exp OR 'parent'/exp OR 'female'/exp OR 'male'/exp OR 'student'/exp

**----**
