# Supplementary material for: Digital Interventions Targeting Healthy and Sustainable Eating Behavior: Systematic Review and Meta-Analysis
Source: J Med Internet Res. 2026 Jan 8;28:e80821. doi: 10.2196/80821 (PMC12782463; doi:10.2196/80821)
Supplement: Multimedia Appendix 2 [file jmir-v28-e80821-s002.pdf]

Multimedia Appendix 3: Risk of Bias

Risk of Bias – RCTs

Author, year	Randomization bias	Intervention bias	Missing outcome bias	Measurement of outcome bias	Selection of reported result bias	Total risk of bias
Brookie et al (2017) [73]	moderate	low	low	moderate	low	**moderate**
Buller et al (2008) [60]	low	low	low	moderate	moderate	**moderate**
Buller et al (2009) [97]	moderate	low	low	moderate	moderate	**moderate**
Carfora et al (2017) [68]	low	low	low	moderate	moderate	**moderate**
Carfora et al (2017) [69]	low	low	low	moderate	moderate	**moderate**
Carfora and Cattelani (2023) [64]	low	low	low	moderate	moderate	**moderate**
Dumas et al (2020) [78]	low	low	low	low	moderate	**moderate**
Elbert et al (2016) [92]	low	low	low	low	moderate	**moderate**
Frie et al (2022) [52]	low	low	low	moderate	low	**moderate**
Kilb et al (2023) – study 1 [48]	moderate	moderate	low	moderate	moderate	**high**
Kilb et al (2023) – study 2 [48]	moderate	low	low	moderate	low	**moderate**
Kothe and Mullan (2014) [99]	low	low	low	moderate	moderate	**moderate**
Lim et al (2021) [63]	moderate	low	moderate	moderate	moderate	**high**
Meng et al (2017) [84]	low	low	low	low	moderate	**moderate**
Nakamura et al (2017) [62]	moderate	low	moderate	moderate	moderate	**high**
Plaete et al (2015) [51]	moderate	low	moderate	moderate	moderate	**high**
Ragelienė et al (2021) [93]	moderate	low	moderate	moderate	moderate	**high**
Røed et al (2021) [80]	low	low	low	low	low	**low**
Rompotis et al (2014) [74]	low	low	low	moderate	moderate	**moderate**
Shatwan et al (2023) [94]	low	moderate	moderate	low	moderate	**moderate**
Springvloet et al (2015) [56]	low	low	moderate	low	low	**moderate**
Springvloet et al (2015) [57]	low	low	moderate	low	low	**moderate**

Tapper et al (2014) [79]	low	low	low	low	moderate	**moderate**
Weber and Nigg (2022) [58]	moderate	low	low	moderate	moderate	**moderate**
Carfora et al (2019) [70]	low	low	low	low	moderate	**moderate**
Carfora et al (2019) [67]	low	low	low	moderate	moderate	**moderate**
Carfora et al (2016) [116]	low	low	low	moderate	moderate	**moderate**
Carfora et al (2022) [65]	low	low	low	low	moderate	**moderate**
Carfora et al (2022) [71]	low	low	low	moderate	moderate	**moderate**
Inauen et al (2017) [86]	low	low	low	moderate	low	**moderate**
Thompson et al (2015) [96]	low	low	low	low	low	**low**
Wolstenholme et al (2020) [72]	low	low	low	moderate	moderate	**moderate**
Carreño et al (2024) [88]	low	low	moderate	moderate	low	**moderate**
Hawkins et al (2024) [134]	low	low	low	moderate	moderate	**moderate**
Liu et al (2025) [91]	low	low	low	low	low	**low**
Livingstone et al (2025) [82]	low	low	low	moderate	low	**moderate**

Risk of Bias - cluster RCTs

**Author, year**	**Randomization bias**	**Bias from timing of identification or recruitment of participants**	**Intervention bias**	**Missing outcome bias**	**Measurement of outcome bias**	**Selection of reported result bias**	**Total risk of bias**
Chamberland et al (2017) [61]	moderate	low	low	low	moderate	moderate	moderate
Gustafson et al (2019) [75]	moderate	low	low	low	moderate	moderate	moderate
Pedersen et al (2016) [76]	moderate	moderate	low	low	moderate	moderate	moderate

Risk of Bias - non-randomized trials

**Author, year**	**Bias due to confounding**	**Bias in selection of participants into the study**	**Bias in classification of interventions**	**Bias due to deviations from intended interventions**	**Missing outcome bias**	**Measurement of outcome bias**	**Selection of reported result bias**	**Total risk of bias**
Alexander et al (2010) [77]	low	low	low	low	low	moderate	moderate	**moderate**
Block et al (2004) [98]	moderate	low	high	low	moderate	moderate	moderate	**high**
Gosliner et al (2023) [66]	moderate	low	low	low	high	moderate	moderate	**high**
Hendrie et al (2020) [89]	low	moderate	low	low	moderate	moderate	moderate	**moderate**
Ng et al (2022) [87]	moderate	low	low	low	low	moderate	moderate	**moderate**
Papadaki and Scott (2005) [54]	moderate	moderate	low	low	low	moderate	moderate	**moderate**
Papadaki and Scott (2008) [55]	moderate	moderate	low	low	moderate	moderate	moderate	**high**
Plaete et al (2016) [51]	low	moderate	low	low	high	moderate	low	**moderate**
Power and Bersamin (2018) [59]	moderate	low	low	low	low	moderate	moderate	**moderate**
Stewart et al (2022) [53]	low	low	low	low	moderate	moderate	low	**moderate**
Vázquez-Paz et al (2022) [90]	high	low	low	low	low	moderate	moderate	**high**
Espinosa-Curiel et al (2020) [95]	moderate	low	low	low	low	low	moderate	**moderate**
Eckert et al (2025) [81]	high	moderate	low	low	moderate	moderate	moderate	**high**
Ricci et al (2025) [83]	moderate	moderate	moderate	low	moderate	moderate	moderate	**high**
