# Supplementary material for: Digital Interventions Targeting Healthy and Sustainable Eating Behavior: Systematic Review and Meta-Analysis
Source: J Med Internet Res. 2026 Jan 8;28:e80821. doi: 10.2196/80821 (PMC12782463; doi:10.2196/80821)
Supplement: Multimedia Appendix 3 [file jmir-v28-e80821-s003.docx]

|  | BCT present | | | | BCT absent | | | Test of moderators | |
| --- | --- | --- | --- | --- | --- | --- | --- | --- | --- |
| BCT | k | *d* | 95% CI | | k | *d* | 95% CI | Qm p-value | |
| 1.1 Goal setting (behaviour)  1.2 Problem solving  1.4 Action planning  1.5 Review behavioural goal  1.6 Discrepancy  1.9 Commitment  2.2 Feedback on behaviour  2.3 Self-monitoring  3.1 Social support  4.1 Instructions how to perform  5.1 Health consequences  5.3 Environmental consequences  5.5 Anticipated regret  5.6 Emotional consequences  6.1 Demonstration  6.2 Social comparison  6.3 Others’ approval  7.1 Prompts/cues  8.1 Practice/rehearsal  8.2 Behaviour substitution  8.3 Habit formation  9.1 Credible source  10.2 Material reward  10.4 Social reward  12.1 Restructuring environment  13.4 Valued self-identify  15.1 Persuasion capability  15.4 Self-talk | 35  13  16  7  8  6  19  27  12  28  28  23  3  5  12  17  7  6  4  6  3  7  5  6  6  3  3  3 | 0.33***  0.24*  0.34*  0.23*  0.44  0.32  0.34***  0.32***  0.38**  0.25**  0.27***  0.32***  0.26  0.39*  0.40*  0.33**  0.45  0.58  0.29  0.34  0.38  0.49  0.25  0.38*  0.33*  0.25  0.33  0.25 | 0.20-0.46  0.06-0.43  0.09-0.58  0.01-0.44  0.00-0.88  -0.30-0.94  0.18-0.49  0.17-0.48  0.18-0.58  0.10-0.39  0.17-0.36  0.20-0.45  -0.15-0.66  0.10-0.68  0.02-0.79  0.17-0.50  -0.15-1.04  -0.41-1.58  -0.75-1.32  -0.06-0.74  -0.11-0.87  -0.06-1.03  -0.18-0.56  0.04-0.72  0.11-0.55  -0.03-0.55  -5.22-5.88  -0.04-0.55 | 22  44  41  50  49  51  38  30  45  29  29  34  54  52  45  40  50  47  53  47  54  50  52  47  51  54  54  54 | | 0.33***  0.36***  0.33***  0.35***  0.31***  0.33***  0.33***  0.34***  0.32***  0.40***  0.39***  0.34***  0.34***  0.33***  0.32***  0.33***  0.32***  0.31***  0.34***  0.33***  0.33***  0.31***  0.34***  0.32***  0.33***  0.33***  0.33***  0.34*** | 0.22-0.45  0.26-0.46  0.23-0.43  0.25-0.44  0.23-0.40  0.24-0.42  0.23-0.42  0.24-0.44  0.21-0.42  0.29-0.52  0.26-0.52  0.22-0.45  0.25-0.43  0.23-0.42  0.23-0.40  0.23-0.43  0.23-0.41  0.23-0.39  0.25-0.42  0.24-0.42  0.24-0.42  0.23-0.39  0.25-0.43  0.23-0.42  0.24-0.42  0.25-0.42  0.25-0.42  0.25-0.42 | | 1.00  .23  .96  .32  .30  .94  .94  .84  .54  .07  .10  .88  .61  .63  .44  .97  .39  **.05**  .78  .95  .76  .15  .44  .65  .97  .71  .99  .71 |

**P*<.05, ***P*<.01, ****P*<.001
