# Supplementary material for: Digital Interventions Targeting Healthy and Sustainable Eating Behavior: Systematic Review and Meta-Analysis
Source: J Med Internet Res. 2026 Jan 8;28:e80821. doi: 10.2196/80821 (PMC12782463; doi:10.2196/80821)
Supplement: Multimedia Appendix 4 [file jmir-v28-e80821-s004.docx]

**Multimedia Appendix 4:** Calculation of effect sizes and meta-analysis

For quantitative synthesis, standardized mean difference effect sizes (Cohen’s *d*) were calculated using the means, standard deviations and sample sizes (pooled standard deviations). For pre-post (within-subject) designs, Cohen’s *dav* was estimated, which is based on the mean difference and average standard deviation of both sets of observations [100,101]. Calculating the variance of Cohen’s *dav* requires knowledge of the correlation (*r*) between measures. As *r* is often not reported in studies, an alternative calculation was used based on the variance of independent samples [114]. For studies that included both an intervention and control group, several measures of effect size are available. In this review, we calculated Cohen’s d based on the mean pre-post change in the treatment group minus the mean pre-post change in the control group, divided by the pooled pretest standard deviation [102]. This estimation has proven to be the most recommended effect size for repeated measure designs in terms of bias and precision [103]. The variance of this effect size was calculated in R with equation 25 of Morris [103], assuming a standard pre-to postintervention correlation of *r*=0.50. Sensitivity analyses were conducted with correlation values of *r*=0.10 and *r*=0.90, with no differences results identified.

Robust Variance Estimation (RVE) was used to calculate a Correlated and Hierarchical Effects Model [106,107]. For data with a complex dependence structure, this method offers a robust solution by grouping effect sizes based on commonalities (i.e. hierarchical clustering) and accounting for the correlation of sampling errors (as some effect sizes stem from the same study sample). However, as this complex dependency of data hinders the understandability of this review, the effect sizes within one study of multiple types of food outcome were aggregated (e.g. if vegetable intake and fruit intake were measured separately, this was aggregated to FVI) [105]. As an estimate of rho (i.e. the correlation of effect sizes) is required to develop efficient weights, several sensitivity analyses were conducted which led to the standard size of *r*=0.5 [104]. Six effect sizes were outliers of which five had a value above *d*=3 and one had a value of *d*=1.62, and were therefore dropped from the meta-analysis [50,51,96]. Moreover, calculation of the standardized mean difference of Dumas et al. [78] led to a medium to strong effect size due to a strong change in fruit and vegetable intake compared to the control group, while the study reported insignificance. As these results did not match up, this study was dropped from the meta-analysis. Eventually, 41 studies were included in the meta-analysis, with a total of 82 effect sizes.

1. Plaete J, Crombez G, Van der Mispel C, Verloigne M, Van Stappen V, De Bourdeaudhuij I. Effect of the web-based intervention MyPlan 1.0 on self-reported fruit and vegetable intake in adults who visit general practice: a quasi-experimental trial. J Med Internet Res. Feb 29, 2016;18(2):e47. [doi: 10.2196/jmir.5252] [Medline: 26929095]
2. Plaete J, De Bourdeaudhuij I, Verloigne M, Crombez G. Acceptability, feasibility and effectiveness of an eHealth behaviour intervention using self-regulation: “MyPlan”. Patient Educ Couns. Jul 26, 2015;98(12):1617-1624. [doi: 10.1016/j.pec.2015.07.014] [Medline: 26277282]
3. Dumas AA, Lemieux S, Lapointe A, Provencher V, Robitaille J, Desroches S. Effects of an evidence-informed healthy eating blog on dietary intakes and food-related behaviors of mothers of preschool- and school-aged children: a randomized controlled trial. J Acad Nutr Diet. Jan 2020;120(1):53-68. [doi: 10.1016/j.jand.2019.05.016] [Medline: 31519466]
4. Thompson D, Bhatt R, Vazquez I, et al. Creating action plans in a serious video game increases and maintains child fruit-vegetable intake: a randomized controlled trial. Int J Behav Nutr Phys Act. Dec 2015;12(1). [doi: 10.1186/s12966-015-0199-z]
5. Cumming G. Understanding the New Statistics: Effect Sizes, Confidence Intervals, and Meta-Analysis. Routledge; 2013. [doi: 10.4324/9780203807002]
6. Lakens D. Calculating and reporting effect sizes to facilitate cumulative science: a practical primer for t-tests and ANOVAs. Front Psychol. Nov 26, 2013;4:863. [doi: 10.3389/fpsyg.2013.00863] [Medline: 24324449]
7. Lenhard W. Computation of different effect sizes like d, f, r and transformation of different effect sizes. Psychometrica. URL: https://www.psychometrica.de/effect_size.html [Accessed 2025-12-17]
8. Morris SB. Estimating Effect Sizes From Pretest-Posttest-Control Group Designs. Organ Res Methods. Apr 2008;11(2):364-386. [doi: 10.1177/1094428106291059]
9. Hedges LV, Tipton E, Johnson MC. Robust variance estimation in meta-regression with dependent effect size estimates. Res Synth Methods. Jan 2010;1(1):39-65. [doi: 10.1002/jrsm.5] [Medline: 26056092]
10. Borenstein M, Hedges LV, Higgins JPT, Rothstein HR. A basic introduction to fixed-effect and random-effects models for meta-analysis. Res Synth Methods. Apr 2010;1(2):97-111. [doi: 10.1002/jrsm.12] [Medline: 26061376]
11. Harrer M, Cuijpers P, Furukawa TA, Ebert DD. Doing Meta-Analysis with R: A Hands-on Guide. Chapman & Hall; URL: https://www.routledge.com/Doing-Meta-Analysis-with-R-A-Hands-On-Guide/Harrer-Cuijpers-Furukawa-Ebert/p/book/9780367610074
12. Pustejovsky JE, Tipton E. Meta-analysis with robust variance estimation: expanding the range of working models. Prev Sci. Apr 2022;23(3):425-438. [doi: 10.1007/s11121-021-01246-3] [Medline: 33961175]
13. Hirst RJ, Cragg L, Allen HA. Vision dominates audition in adults but not children: a meta-analysis of the Colavita effect. Neurosci Biobehav Rev*.* 2018;94:286-301 [doi: 10.1016/j.neubiorev.2018.07.012]
