# Supplementary material for: Digital Interventions Targeting Healthy and Sustainable Eating Behavior: Systematic Review and Meta-Analysis
Source: J Med Internet Res. 2026 Jan 8;28:e80821. doi: 10.2196/80821 (PMC12782463; doi:10.2196/80821)
Supplement: Multimedia Appendix 6 [file jmir-v28-e80821-s006.docx]

| BCTs | k | % |
| --- | --- | --- |
| **1. Goals and planning**  1.1 Goal setting (behavior)  1.2 Problem solving  1.4 Action planning  1.5 Review behavioral goals  1.6 Discrepancy between current behavior and goal  1.7 Review outcome goal(s)  1.9 Commitment  **2. Feedback and monitoring**  2.1 Monitoring of behavior by others without feedback  2.2 Feedback on behavior  2.3 Self-monitoring of behavior  **3. Social support**  3.1 Social support (unspecified)  3.3 Social support (emotional)  **4. Shaping knowledge**  4.1 Instruction on how to perform the behavior  4.2 Information about antecedents  **5. Natural consequences**  5.1 Information about health consequences  5.2 Salience of consequences  5.3 Information about social and environmental consequences  5.4 Monitoring of emotional consequences  5.5 Anticipated regret  5.6 Information about emotional consequences  **6. Comparison of behavior**  6.1 Demonstration of the behavior  6.2 Social comparison  6.3 Information about others’ approval  **7. Associations**  7.1 Prompts/cues  **8. Associations**  8.1 Behavioral practice/rehearsal  8.2 Behavior substitution  8.3 Habit formation  8.4 Habit reversal  8.7 Graded tasks  **9. Comparison of outcomes**  9.1 Credible source  9.2 Pros and cons  9.3 Comparative imagining of future outcomes **10. Reward and threat**  10.2 Material reward (behavior)  10.3 Non-specific reward  10.4 Social reward  10.6 Non-specific incentive  **12. Antecedents**  12.1 Restructuring the physical environment  12.3 Avoidance/reducing exposure to cues for the behavior  12.6 Body changes  **13. Identity**  13.1 Identification of self as role model 13.2 Framing/reframing  13.3 Incompatible beliefs  13.4 Valued self-identify  **14. Scheduled consequences** 14.4 Reward approximation  14.5 Reward completion 14.7 Reward incompatible behavior 14.9 Reduce reward frequency **15. Self-belief**  15.1 Verbal persuasion about capability  15.2 Mental rehearsal of successful performance  15.3 Focus on past success  15.4 Self-talk  **16. Covert learning**  16.2. Imaginary reward  16.3 Vicarious consequences | **45**  41  15  15  8  8  1  6  **36**  1  23  26  **16**  14  2  **29**  29  1  **44**  33  1  25  1  3  6  **28**  13  17  6  **8**  8  **16**  6  8  5  1  5  **13**  10  4  1  **15**  6  6  8  2  **8**  8  1  1  **11**  4  2  3  3  **4**  2  2  2  1  **9**  5  1  1  3  **4**  3  1 | **65.2%**  59.4%  21.7%  21.7%  11.6%  11.6%  1.4%  8.7%  **52.2%**  1.4%  33.3%  37.7%  **23.2%**  20.3%  2.9%  **42.0%**  42.0%  1.4%  **63.8%**  47.8%  1.4%  36.2%  1.4%  4.3%  8.7%  40.6%  18.8%  24.6%  8.7%  **11.6%**  11.6%  **23.2%**  8.7%  11.6%  7.2%  1.4%  7.2%  **18.8%**  14.5%  5.8%  1.4%  **21.7%**  8.7%  8.7%  11.6%  2.9%  **11.6%**  11.6%  1.4%  1.4%  **15.9%**  5.8%  2.9%  4.3%  4.3%  **5.8%**  2.9%  2.9%  2.9%  1.4%  **13.0%**  7.2%  1.4%  1.4%  4.3%  **5.8%**  4.3%  1.4% |
