# Supplementary material for: Digital Interventions Targeting Healthy and Sustainable Eating Behavior: Systematic Review and Meta-Analysis
Source: J Med Internet Res. 2026 Jan 8;28:e80821. doi: 10.2196/80821 (PMC12782463; doi:10.2196/80821)
Supplement: Multimedia Appendix 7 [file jmir-v28-e80821-s007.pdf]

Figure S1. Graphical representation of outliers

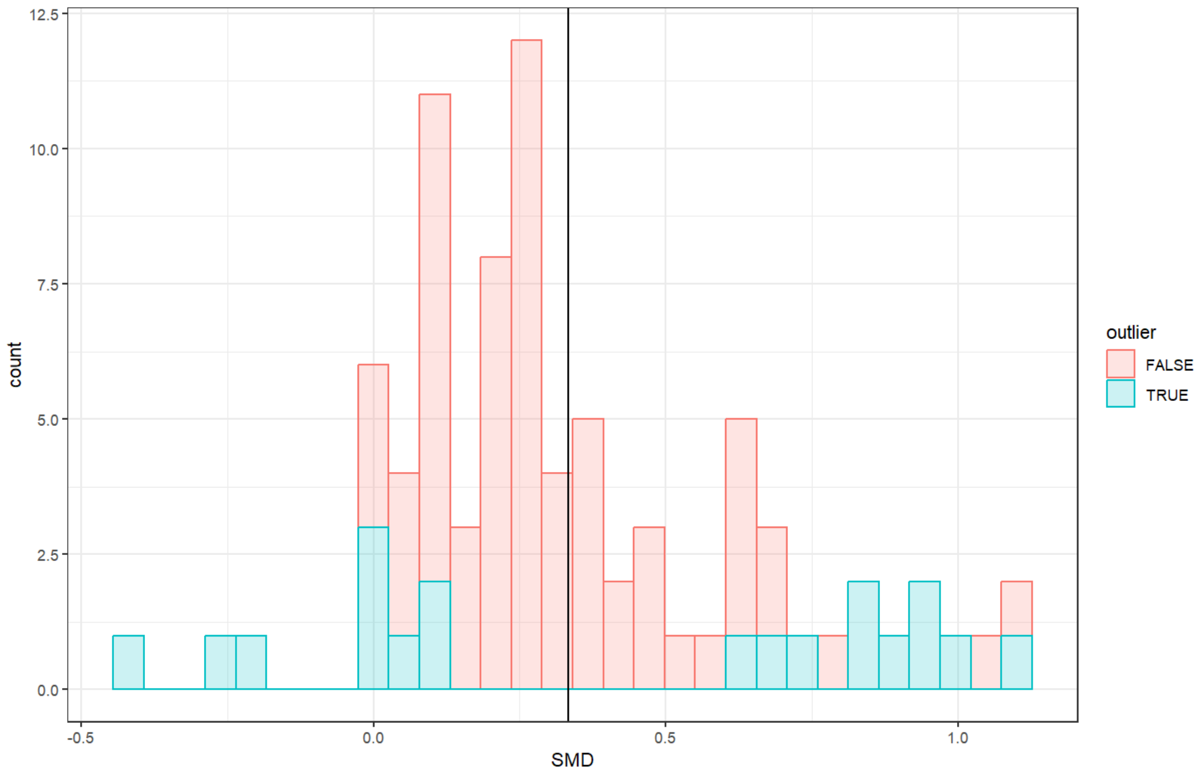

Figure S2. Funnel plot

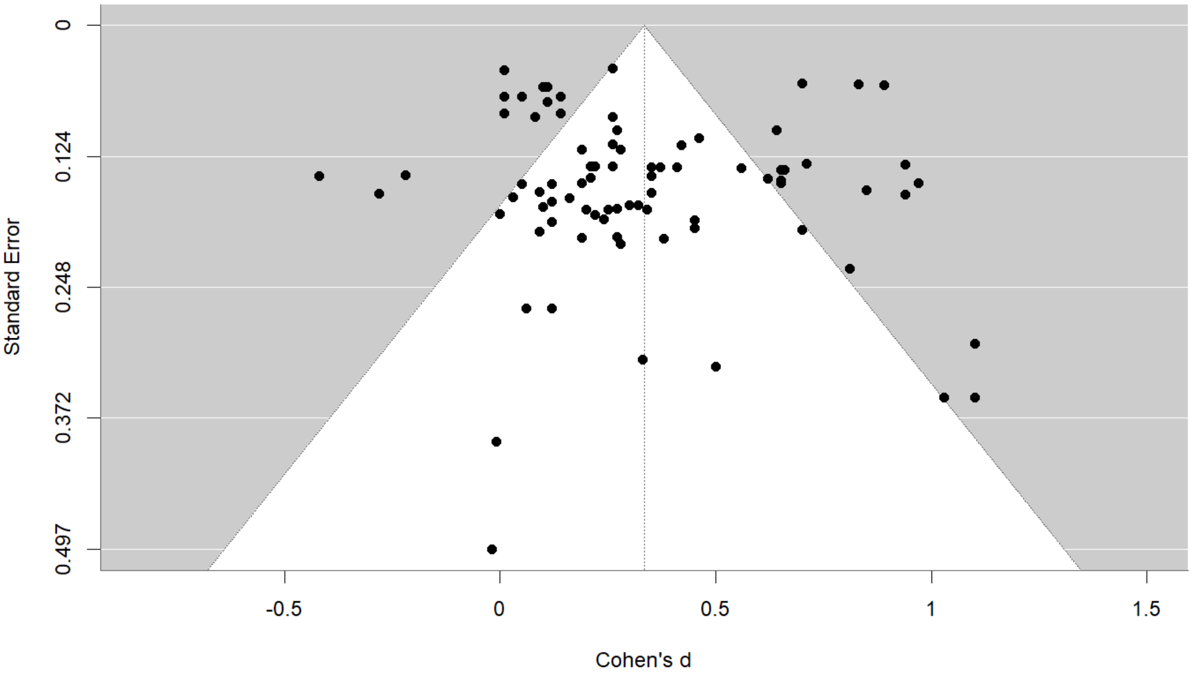

Table S1. General sensitivity analyses

**General meta-analysis**	*d*	95% CI	I^2	95% PI
without potential outliers	0.29***	0.24 – 0.34	41%	0.07 – 0.51
without high-risk-of-bias studies	0.36***	0.27 – 0.45	86%	-0.21 – 0.93

Table S2. Sensitivity analyses excluding non-randomized studies: general meta-analysis and subgroup analyses

**Analyses without NRS**	*d*	95% CI	I^2	95% PI
General meta-analysis	0.32***	0.23 – 0.41	84%	-0.23 – 0.87
**Subgroup – Time measure** (*P*=.73)				
Post-intervention	0.31***	0.23 – 0.40	65%	-0.19 – 0.82
Follow-up	0.34**	0.19 – 0.48	76%	-0.29 – 0.83
**Subgroup – Goal orientation** (*P*=.64*)*				
Promotion focus	0.32***	0.22 – 0.42	74%	-0.23 – 0.88
Prevention focus	0.33***	0.14 – 0.53	64%	-0.09 – 0.76
**Subgroup – Age group** (*P*=.08)				
Adults	0.25**	0.10 – 0.40	62%	-0.21 – 0.71
Young adults	0.46***	0.30 – 0.61	65%	-0.10 – 1.00
Adolescents	0.18	-0.06 – 0.42	86%	-0.45 – 0.84
**Subgroup – Digital medium** (*P*=.01)				
Text messaging	0.38***	0.25 – 0.50	58%	0.00 – 0.73
Website	0.23**	0.09 – 0.38	73%	-0.25 – 0.72
Social media	0.63***	0.37 – 0.88	52%	-0.12 – 1.37
Mobile app	NA	NA	NA	NA

****P*<.001, ***P*<.01

Analyses without NRS: Pairwise comparison subgroups - Digital medium

Social media vs. text messaging (*t*(66)= 1.72, *P*=.09)

Social media vs. website (*t*(66)= 2.61, *P*=.01)

Table S3. Sensitivity analyses excluding non-randomized studies: subgroup analyses with BCT cluster as moderator.

	BCT cluster present			BCT cluster absent			Test of moderators
BCT cluster	k	*d*	95% CI	k	*d*	95% CI	Qm P-value
1. Goals and planning	32	0.29***	0.16-0.42	18	0.36***	0.24-0.48	.46
2. Feedback and monitoring	28	0.29***	0.15-0.44	22	0.34***	0.23-0.46	.58
3. Social support	14	0.36***	0.16-0.55	36	0.30***	0.21-0.40	.56
4. Shaping knowledge	23	0.24***	0.07-0.40	27	0.38***	0.26-0.50	.13
5. Natural consequences	32	0.27***	0.19-0.35	18	0.38**	0.18-0.60	.21
6. Comparison of behavior	25	0.38***	0.22-0.54	25	0.27***	0.18-0.35	.15
7. Associations	5	0.62	-2.57-3.80	45	0.31***	0.23-0.39	.04
8. Repetition and substitution	9	0.25**	0.07-0.43	41	0.33***	0.23-0.43	.41
9. Comparison of outcomes	6	0.40**	-0.74-1.54	44	0.30***	0.22-0.39	.49
10. Reward and threat	10	0.31**	0.04-0.57	40	0.32***	0.21-0.42	.94
12. Antecedents	6	0.32**	0.10-0.54	44	0.32***	0.22-0.41	.97
13. Identity	8	0.17*	0.03-0.31	42	0.35***	0.25-0.45	.10
14. Scheduled consequences	2	NA	NA	48	0.33***	0.23-0.42	NA
15. Self-belief	6	0.21	-0.05-0.47	45	0.33***	0.23-0.43	.41

**P*<.05, ***P*<.01, ****P*<.001
